# Targeting the Gut Microbiota in Pediatric Obesity: A Paradigm Shift in Prevention and Treatment? A Comprehensive Review

**DOI:** 10.3390/nu17182942

**Published:** 2025-09-12

**Authors:** Gianmario Forcina, Pierluigi Di Filippo, Delia De Biasio, Francesco Giustino Cesaro, Vittoria Frattolillo, Alessia Massa, Maria De Cesare, Pierluigi Marzuillo, Emanuele Miraglia del Giudice, Anna Di Sessa

**Affiliations:** Department of Woman, Child, and General and Specialized Surgery, University of Campania “Luigi Vanvitelli”, 80138 Naples, Italy; gianmario.forcina@gmail.com (G.F.); pierluigi.difilippo94@gmail.com (P.D.F.); deliadebiasio@libero.it (D.D.B.); francescocesaro1993@gmail.com (F.G.C.); vitto.fratt@gmail.com (V.F.); alessiamassax@gmail.com (A.M.); maria.decesare91@gmail.com (M.D.C.); pierluigi.marzuillo@unicampania.it (P.M.); emanuele.miraglia@unicampania.it (E.M.d.G.)

**Keywords:** pediatric obesity, gut microbiota, dysbiosis, targeted interventions

## Abstract

Pediatric obesity represents a growing global health challenge, closely associated with increased cardiometabolic risk and long-term adverse outcomes. Although lifestyle modifications remain the cornerstone of treatment, sustained success is often limited. Emerging evidence suggests that the gut microbiota (GM) plays a pivotal role in the pathogenesis of obesity, influencing host metabolism, energy homeostasis, and systemic inflammation. This narrative review aims to provide a comprehensive and up-to-date overview of the complex interplay between GM and pediatric obesity, with a particular emphasis on microbiota-targeted interventions. These include probiotics, prebiotics, synbiotics, postbiotics, dietary modulation, and fecal microbiota transplantation (FMT). Findings from preclinical studies and early-phase clinical trials indicate that gut dysbiosis may contribute to obesity-related mechanisms, such as altered nutrient absorption, increased adiposity, and dysregulated appetite control. Interventions targeting the microbiota have shown promise in modulating inflammatory pathways and improving metabolic profiles. While preliminary findings underscore the potential of the GM as a novel adjunctive target in managing pediatric obesity, current evidence remains heterogeneous, and robust clinical pediatric data are limited. Further research is needed to clarify the therapeutic efficacy, safety, and long-term outcomes of microbiota-modulating strategies in children with obesity.

## 1. Introduction

In recent years, pediatric obesity has reached epidemic proportions globally, emerging as one of the most significant public health challenges worldwide [1,2].

Recent estimates indicate that the global prevalence of pediatric obesity has nearly tripled over the past four decades [2,3], with 124 million children aged 5–19 years affected by obesity and over 213 million classified as overweight in 2016 [4,5]. Post-pandemic data suggest a further increase, with a reported global average prevalence of obesity among children and adolescents at 8.5% in 2023 [6].

Pediatric obesity is increasingly recognized as a complex and multifactorial condition, influenced by both genetic predispositions and environmental factors, including socioeconomic determinants, unhealthy dietary habits, and sedentary lifestyles [7,8,9]. Of particular concern, obesity is associated with early-onset metabolic abnormalities such as insulin resistance (IR), dyslipidemia, and low-grade systemic inflammation, which significantly elevate the risk of long-term comorbidities, particularly cardiovascular, renal, hepatic, and metabolic diseases [10,11,12,13,14].

Despite growing awareness and clinical urgency, current therapeutic strategies for pediatric obesity remain largely focused on lifestyle interventions, including nutritional education, increased physical activity, and behavioral modification [15,16,17,18]. However, these approaches often suffer from poor adherence and sustainability, with effectiveness varying considerably depending on individual characteristics, socioeconomic status, and family background [15].

Pharmacological treatments for pediatric obesity are still limited, with only a few options approved for severe or comorbid obesity [15]. Among these, metformin is commonly used off-label in young patients with obesity and IR [19,20,21]. However, the most promising pharmacotherapies are glucagon-like peptide-1 (GLP-1) receptor agonists, such as liraglutide and semaglutide [22,23].

Additionally, bariatric surgery is reserved for adolescents meeting strict clinical criteria [24,25]. Current guidelines recommend bariatric surgery in adolescents with a body mass index (BMI ≥ 35) kg/m^2^ (or ≥120% of the 95th percentile) and at least one serious obesity-related comorbidity, such as type 2 diabetes (T2D), moderate-to-severe obstructive sleep apnea (OSA), hypertension, or nonalcoholic steatohepatitis (NASH) with fibrosis, or with a BMI ≥ 40 kg/m^2^ (or ≥140% of the 95th percentile) irrespective of comorbidities, provided that candidates are pubertally mature, psychosocially stable, and followed by a multidisciplinary team [24,25].

These gaps highlight the urgent need for novel, safe, and modifiable therapeutic targets, particularly in pediatric populations where long-term risk mitigation is crucial.

In this evolving landscape, the gut microbiota (GM) has emerged as a promising therapeutic target for pediatric obesity, given its potential pathophysiological link to the disease [26,27,28,29,30]. This broad and complex microbial community—comprising bacteria, viruses, fungi, and archaea, with an estimated total of approximately 10^14^ microorganisms—has become increasingly recognized as a dynamic ecosystem with critical roles in host metabolism and immune system maturation [26,27,31].

As such, the GM is no longer considered merely a biomarker of metabolic status but is increasingly viewed as a modifiable therapeutic axis, with the potential to reshape the natural history of diseases [31,32,33,34].

Consequently, several strategies have been proposed to manipulate GM, including dietary interventions, probiotics, prebiotics, synbiotics, postbiotics, and fecal microbiota transplantation (FMT) [33,34]. Among these, probiotics have gained particular attention, since they may exert beneficial effects through modulation of short-chain fatty acids (SCFAs) and bile acid signaling, enhancement of gut barrier function, reduction of systemic inflammation, and interaction with the gut–brain axis influencing appetite and insulin sensitivity [32,33,34].

This review aims to provide a comprehensive overview of the emerging role of GM as a preventive and therapeutic target for personalized intervention strategies aimed at mitigating long-term cardiometabolic risk in children with obesity. By incorporating recent findings on personalized intervention strategies and highlighting the unique characteristics and limitations of pediatric populations, we aim to provide new insights and methodological perspectives that go beyond the existing literature in this evolving field.

## 2. Methods

A comprehensive literature search was conducted across major databases, including PubMed, Medline, Scopus, Web of Science, and Google Scholar, to identify relevant studies published up to August 2025. Additionally, reference lists of key articles and systematic reviews were manually screened to capture pertinent studies potentially missed during the initial search.

Keywords such as “gut microbiota modulation,” “children,” “obesity,” “targeted interventions,” “treatment,” and “prevention” were used in various combinations. Studies were selected according to predefined inclusion and exclusion criteria. Eligible publications included peer-reviewed articles in English involving children and adolescents (0–18 years) that examined associations between gut microbiota and obesity or interventions targeting the microbiota to influence metabolic or weight-related outcomes. Considered study types encompassed original research, clinical trials, systematic reviews, and meta-analyses. Excluded were non-English publications, non-peer-reviewed articles, case reports, studies with insufficient data, and unpublished reports.

Articles were screened for relevance and scientific contribution following the PRISMA guidelines. Key data were manually extracted and synthesized descriptively.

Consistent with the narrative review approach, no formal quality assessment or meta-analysis was conducted.

Notably, potential publication bias—stemming from the selective inclusion of published studies and exclusion of unpublished data—may limit the reliability and generalizability of the findings.

## 3. The Role of GM in Pediatric Obesity

**Figure 1:**
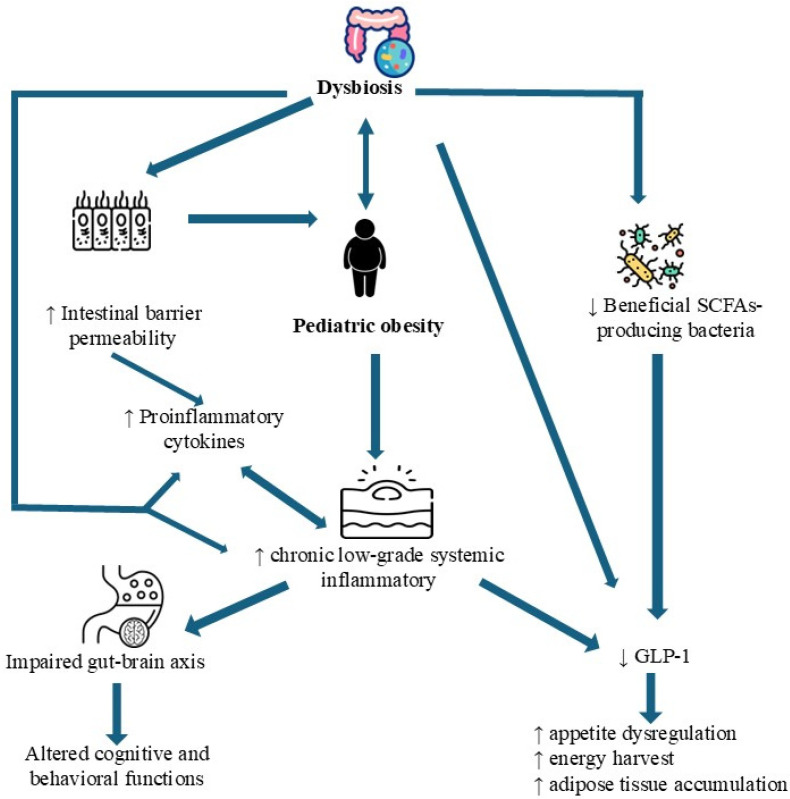
The complex pathophysiological interplay between gut dysbiosis and pediatric obesity: inflammatory, endocrine, and neurobehavioral pathways. Abbreviations: GLP-1: Glucagon-like peptide-1; SCFAs: Short-Chain Fatty Acids.

The GM constitutes a highly complex and dynamic microbial ecosystem, comprising trillions of microorganisms that contribute critically to numerous physiological processes, including digestion, maturation of the immune system, endocrine signaling, and regulation of neurobehavioral functions [35] (Figure 1).

During early life, the GM is highly susceptible to external influences, including mode of delivery, infant feeding practices, antibiotic exposure, and maternal diet [35,36,37]. Disruptions to the GM during this critical developmental window can result in dysbiosis—an imbalance in microbial composition—which has been increasingly linked to pediatric obesity. This association extends beyond energy extraction and fat accumulation, encompassing immune, endocrine, neurobehavioral, and epigenetic mechanisms [28,38]. However, although emerging evidence suggests that gut dysbiosis may contribute to obesity through mechanisms such as endotoxemia, inflammation, and hormonal alterations, a direct causal relationship in pediatric populations remains to be clearly demonstrated [26,27,28,29].

A major consequence of dysbiosis is the compromise of intestinal barrier integrity, leading to enhanced gut permeability. This disruption permits the translocation of microbial components such as lipopolysaccharide (LPS) into the systemic circulation, thereby triggering low-grade inflammation and metabolic disturbances [28,39]. This condition, termed metabolic endotoxemia, activates Toll-like receptor 4 (TLR4) and its downstream signaling pathways, perpetuating chronic systemic inflammation [28,39]. Concurrently, dysbiosis impairs immune regulation by diminishing regulatory T cell populations and promoting pro-inflammatory cytokine release, further amplifying metabolic dysfunction [28,39].

Endocrine dysregulation also plays a pivotal role in the dysbiosis–obesity connection. The GM influences both the secretion and sensitivity of key hormones, including insulin, leptin, ghrelin, glucagon-like peptide-1 (GLP-1), and peptide YY (PYY), which collectively modulate appetite, glucose metabolism, and fat storage [39,40,41]. Additionally, the gut–brain axis, a bidirectional communication system involving microbial metabolites and neurotransmitters, critically affects mood, satiety, and behavioral patterns [38]. In dysbiotic states, alterations in serotonin and GABA signaling have been proposed to affect emotional eating behaviors and satiety regulation, although this remains to be fully elucidated, particularly within the context of pediatric obesity [38,42,43].

Epigenetic modifications further underpin the long-term effects of early microbial exposures on metabolic health [44,45,46]. Diet-induced alterations in maternal GM can shape fetal DNA methylation patterns, particularly in genes related to adipogenesis, insulin signaling, and energy regulation [41,46,47]. Moreover, microbial metabolites such as short-chain fatty acids (SCFAs) may serve as epigenetic regulators by modulating DNA methyltransferase and histone deacetylase activity [44,46,48]. Although primarily studied in adults, these epigenetic effects are believed to originate early in life and are influenced by variables such as birth mode, breastfeeding, and environmental exposures, all of which contribute to sustained obesity risk [38,46,49].

The GM has increasingly been recognized as a central regulator of human physiology, serving as a multifaceted interface among the endocrine, metabolic, immune, and nervous systems [35,50,51,52]. It plays essential roles in digestion, vitamin synthesis, immune system development, and maintenance of intestinal barrier function [53,54,55,56].

Beyond these foundational functions, it also modulates energy homeostasis by influencing caloric extraction, bile acid metabolism, and inflammatory processes [39,40]. Additionally, its impact on hormonal pathways further connects it to appetite control, insulin sensitivity, and lipid metabolism [39,40,41].

Altered microbial composition—dysbiosis—has been associated with numerous conditions, including inflammatory bowel disease (IBD), allergies, diabetes (type 1 and 2), neurodevelopmental disorders, metabolic syndrome, and cardiometabolic diseases [56,57,58]. In the context of pediatric obesity, evidence suggests that dysbiosis contributes through disrupted SCFA production, increased systemic inflammation, and altered appetite regulation via the gut–brain axis [56,57,58].

Studies have shown that children with obesity often exhibit reduced microbial diversity and a disrupted *Firmicutes-to-Bacteroidetes* (F/B) ratio, marked by an increased abundance of Firmicutes [39,40,59]. This imbalance promotes bacterial populations associated with inflammation and energy storage. Specifically, a rise in *Firmicutes*, lactobacilli, and *Proteobacteria*, alongside a decrease in beneficial microbes such as *Bifidobacterium*, *Bacteroides*, and *Akkermansia muciniphila*—known for their anti-inflammatory and metabolic roles—has been reported [39,50,51].

This dysbiotic composition compromises intestinal integrity, resulting in increased permeability (commonly referred to as “leaky gut”) [52,56,60]. Consequently, LPS and other bacterial products translocate into the bloodstream, triggering metabolic endotoxemia and reinforcing low-grade inflammation. This process fuels a vicious cycle of metabolic dysfunction and neuroendocrine disruption [52,56,57,60].

These microbial imbalances ultimately contribute to increased fat deposition and IR, reinforcing the obese phenotype [39,40,61]. Given the considerable plasticity of the GM during childhood, disruptions in its early development—whether from cesarean section, formula feeding, or antibiotic use—may significantly elevate the risk of obesity and chronic disease later in life [45,46,62].

Accordingly, targeting the GM represents a promising strategy for the prevention and treatment of pediatric obesity, with emerging interventions aimed at modulating microbial composition and function showing considerable potential [63,64].

## 4. Gut Dysbiosis in Pediatric Obesity

Numerous studies have reported significant alterations in the GM composition of children with obesity compared to their normal-weight peers [65,66,67,68,69]. However, the functional roles of bacterial strains are highly context-dependent and may vary depending on host-specific factors such as age, diet, and comorbidities [65,67,69].

Although its reliability as a marker of obesity remain debated [27,70], a substantial body of evidence supports an increased F/B ratio- commonly associated with enhanced energy extraction and fat accumulation- as a signature for pediatric obesity [67,68,69].

An insightful study revealed that children with obesity exhibited higher levels of lactobacilli and a notable reduction in *Bacteroides vulgatus*. Species-level analysis of the *Bacteroides fragilis* group showed that *B. fragilis* and *B. thetaiotaomicron* were more abundant in the obese group, while *B. caccae*, *B. ovatus*, *B. uniformis*, and especially *B. vulgatus* were more prevalent in lean children. This suggests a compositional shift towards pro-inflammatory and energy-harvesting taxa in children with obesity [65].

Another study investigating obesity-related precocious puberty found that girls with obesity displayed significant differences in GM diversity and composition when compared to healthy controls [71]. Specifically, there was an increased abundance of genera such as *Klebsiella*, *Lachnoclostridium*, *Erysipelotrichaceae UCG-003*, and *Ruminococcus gnavus*, while beneficial bacteria like *Anaerostipes*, *Bifidobacterium*, *Bacteroides*, and *Eubacterium hallii* were depleted. Anaerostipes, notably, was negatively related to key metabolic and hormonal markers, indicating a protective role in both metabolic regulation and pubertal development [71].

A systematic review by Morgado et al. further confirmed that children with obesity typically exhibit a depletion in *Bacteroidetes* and *Bifidobacterium* spp., alongside a reduction in overall microbial diversity [66]. In contrast, their microbiota tends to be enriched in *Proteobacteria*, *Firmicutes*, lactobacilli, *Faecalibacterium*, *Blautia*, *Actinomyces*, *Sutterella*, and *Collinsella* [66].

At the species level, *Bacteroides plebeius*, *Bacteroides dorei*, *Bilophila wadsworthia*, *Clostridium symbiosum*, *Parabacteroides distasonis*, lactobacilli, and *Escherichia coli* were more commonly found in children with obesity. Conversely, lean children showed higher abundances of *B. vulgatus*, *Bifidobacterium* spp., *Oscillospira*, *Dialister*, and *A. muciniphila*, species typically associated with anti-inflammatory activity and gut barrier integrity [66].

Given the compelling role of the GM in regulating metabolic homeostasis, these microbial patterns may serve as potential biomarkers and therapeutic targets for the prevention and treatment of pediatric obesity [64,65,70].

### 4.1. Gut Dysbiosis—Induced Inflammatory and Immune-Metabolic Activation

Systemic inflammation in obesity is characterized by chronic, low-grade immune activation, largely driven by alterations in GM composition and function [67,69,71].

Dysbiosis, which leads to an imbalance in the microbial community, increases intestinal permeability, allowing bacterial products such as LPS to enter the bloodstream. This phenomenon, known as endotoxemia, triggers immune activation [67,72]. LPS acts as a potent ligand for TLR4 on monocytes and macrophages, initiating NF-κB activation and the release of pro-inflammatory cytokines such as TNF-α, IL-1β, and IL-6 [73].

These cytokines drive a shift in macrophage polarization within adipose tissue from the anti-inflammatory M2 phenotype, typically seen in lean individuals, to the pro-inflammatory M1 phenotype [73].

M1 macrophages, along with infiltrating Th1 lymphocytes, neutrophils, dendritic cells, and CD8^+^ T cells, sustain both local and systemic inflammation, further promoting IR and tissue remodeling [74].

Simultaneously, regulatory T cells (Tregs) and anti-inflammatory cytokines (IL-10, TGF-β) are reduced [74,75].

The GM also plays a crucial role in maintaining immune balance through microbial metabolites. Butyrate-producing bacteria such as *Faecalibacterium prausnitzii* and *Roseburia* spp. support Treg expansion and help maintain epithelial barrier integrity [56,76]. In pediatric obesity, the reduction of these beneficial microbes impairs this protective mechanism, perpetuating inflammation and exacerbating metabolic dysfunction [76].

Elevated pro-inflammatory cytokines, particularly TNF-α and IL-6, disrupt insulin signaling by promoting the serine phosphorylation of IRS-1, which interferes with downstream receptor activity and impairs glucose homeostasis [60].

In pediatric populations, this cascade—linking gut dysbiosis, immune dysregulation, and metabolic impairment—may set the stage for lifelong metabolic disease [39].

Modulating the GM-immune system axis represents a promising therapeutic approach for restoring immune tolerance and mitigating obesity-related complications [60,64,74].

### 4.2. Gut Dysbiosis—Driven Hormonal Dysregulation

Gut dysbiosis is increasingly recognized not only as a consequence but also as a significant contributing factor in the pathogenesis of obesity, particularly through its widespread impact on hormonal regulation [57,58,77].

One of the most extensively studied mechanisms involves the overproduction of SCFAs, particularly acetate, propionate, and butyrate, by fermentative gut bacteria [77].

In dysbiotic states, excessive SCFAs production enhances hepatic lipogenesis and gluconeogenesis, promotes insulin and GLP-1 secretion, and disrupts energy homeostasis, contributing to adipose tissue expansion and metabolic overload [57,58,77].

In addition to metabolic alterations, GM modulates the release of appetite-regulating hormones [39,40,41].

A reduced abundance of *Bifidobacterium* and *Bacteroides*, coupled with an increase in *Firmicutes* and *Proteobacteria*, has been associated with elevated ghrelin levels and reduced leptin sensitivity. This imbalance favors persistent hyperphagia and impaired satiety responses [50,51,52]. Such dysregulation leads to leptin resistance—a hallmark of obesity—where, despite elevated leptin levels, hypothalamic receptors fail to respond adequately, promoting excessive caloric intake [50,51,52].

Furthermore, dysbiosis disrupts the intestinal barrier, increasing permeability and facilitating the translocation of bacterial endotoxins, particularly LPS, into the bloodstream. This results in chronic low-grade inflammation and impacts the hypothalamic–pituitary–adrenal (HPA) axis by promoting its activation and elevating cortisol levels [39,60].

Chronic hypercortisolemia not only favors central fat accumulation but also exacerbates IR, while suppressing GLP-1 and PYY secretion, further impairing appetite regulation and glucose homeostasis [39,60].

The gut–brain axis also plays a pivotal role in obesity, as dysbiosis interferes with the synthesis of neurotransmitters, including serotonin, of which 90% is produced in the gut [38,42,43,51,64].

Alterations in serotonin levels, driven by shifts in tryptophan metabolism by the microbiota, can impact mood and appetite regulation, contributing to emotional eating and disrupted satiety signaling—psychobehavioral patterns commonly observed in obesity [51].

Emerging data suggest that GM may also influence sex hormone regulation, particularly through microbial β-glucuronidase activity, which affects estrogen recycling in the gut [39,50].

Dysbiosis may increase circulating estrogen levels and disrupt the hypothalamic-pituitary-gonadal (HPG) axis, contributing to early puberty, particularly in girls with obesity [39,50].

In conclusion, gut dysbiosis contributes to obesity through a complex network of endocrine disruptions, involving insulin, leptin, ghrelin, GLP-1, PYY, cortisol, serotonin, and sex hormones [38,41,42,43].

These hormonal alterations are interrelated and collectively play a critical role in sustaining the obese phenotype [38,42,43].

Within this framework, targeting the GM may represent a promising strategy to restore hormonal balance and promote metabolic improvements [42,77].

## 5. Evidence on the Potential Therapeutic Role of GM in Pediatric Obesity

**Figure 2:**
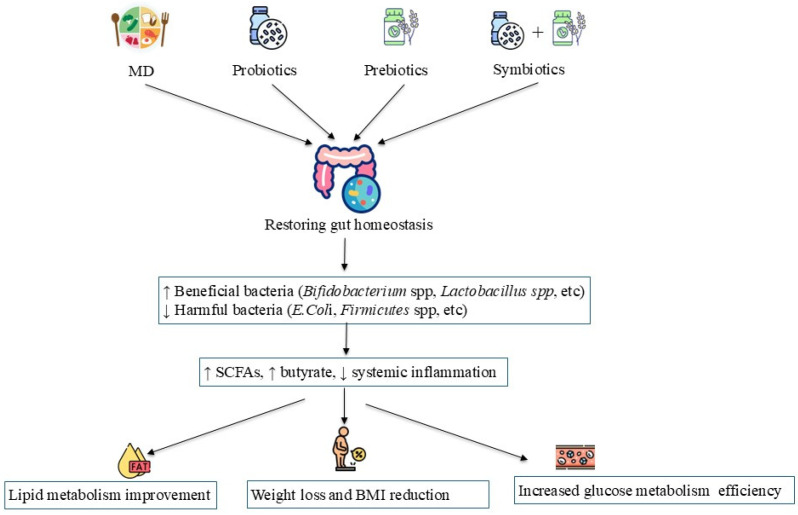
Potential therapeutic role of gut microbiota modulation in pediatric obesity. Abbreviations: BMI: Body mass index; MD: Mediterranean diet; SCFAs: Short-Chain Fatty Acids.

The GM has emerged as a promising therapeutic target in the context of pediatric obesity, with several strategies currently under investigation for their potential to modulate microbial composition and function [78,79,80] (Figure 2).

Among these, the Mediterranean diet (MD) has been shown to enhance microbial diversity and stimulate the production of anti-inflammatory metabolites, contributing to improved metabolic health [78]. Additionally, probiotics, prebiotics, and synbiotics aim to selectively enrich beneficial microbial taxa and modulate host metabolic pathways [79,80].

Fecal microbiota transplantation (FMT) has also garnered interest as a more direct means of reshaping the gut ecosystem [78]. However, its application in pediatric populations remains experimental and requires further clinical validation [78].

### 5.1. MD and GM: A Symbiotic Alliance

The MD, rich in fruits, vegetables, legumes, whole grains, nuts, and extra-virgin olive oil, with moderate fish intake and limited red and processed meats, plays a pivotal role in modulating the GM [81,82]. A central factor in this modulation is its high dietary fiber content, which enhances satiety, supports glycemic control, and provides fermentable substrates for commensal bacteria, benefiting both metabolism and GM function [81,82,83].

Dietary fibers promote the growth of SCFA-producing taxa—such as *Faecalibacte-rium prausnitzii*, *Akkermansia muciniphila*, *Christensenellaceae*, and *Eubacterium rectale*—thereby increasing microbial diversity and gene richness. This enhances SCFA production, particularly propionate and butyrate [83,84,85,86,87], which are associated with improved insulin sensitivity, reduced systemic inflammation, better gut barrier integrity, and appetite regulation via the gut–brain axis [86,88,89]. Propionate, in particular, plays a key role in satiety signaling and energy regulation [90,91]. Greater microbial diversity also supports vital metabolic processes including bile acid transformation, polyphenol metabolism, and Trimethylamine *N*-oxide (TMAO) regulation, all contributing to cardiometabolic health [92].

Adherence to the MD is linked to reduced inflammatory biomarkers such as IL-6, TNF-α, and CRP [92]. These anti-inflammatory effects, together with the promotion of eubiosis, are associated with lower risks of cardiometabolic conditions including obesity, prediabetes, T2D, metabolic-associated fatty liver disease (MASLD), cardiovascular morbidity, and gastrointestinal disorders [93,94,95,96,97].

A meta-analysis by López Gil et al. found that children and adolescents adhering to the MD had significantly lower systemic inflammation, blood pressure, triglycerides, and LDL cholesterol levels [98]. Similarly, Yurtdaş et al. reported that MD adherence in adolescents with NAFLD reduced hepatic steatosis, serum transaminases, IR, and inflammatory markers, while partially restoring GM composition, enhancing SCFAs production, and reducing LPS translocation [99].

In a clinical trial by Dasgupta et al., a 6-month MD-based intervention in school-aged children with prediabetes led to improvements in fasting glycemia, glycated hemoglobin, lipid profile, and inflammatory markers, highlighting the role of microbiota-mediated mechanisms in reducing early T2D risk [100].

Overall, these findings underscore the potential of MD-driven, microbiota-targeted dietary strategies in pediatric populations to reduce systemic inflammation and lower long-term cardiometabolic risk [95,96,97].

### 5.2. Probiotics: Strain-Specific Tools for Pediatric Metabolic Health

Probiotics are live microorganisms that, when properly administered, confer a health benefit on the host [101]. Their role in restoring gut homeostasis and modulating metabolic parameters in children with obesity has been extensively investigated [102,103,104,105,106], though clinical results have shown considerable variability [31,107,108,109] (Table 1).

Probiotics modulate GM composition through multiple mechanisms, including competitive exclusion of pathogenic species, production of beneficial metabolites, and promotion of intestinal beneficial bacteria, such as lactobacilli and *Bifidobacterium* spp. [110,111].

Additionally, probiotics play a crucial role in restoring intestinal epithelial integrity and enhancing the function of the intestinal barrier [110,111]. Therefore, probiotic supplementation has garnered attention as a potential adjunct therapy for pediatric obesity and related metabolic outcomes [102,103,104,105,106]. However, its effectiveness appears to be strain-specific and dose-dependent [107,112,113].

A 2023 double-blind randomized controlled trial (RCT) by Sohn et al. demonstrated the therapeutic potential of *Lactiplantibacillus plantarum* LMT1-48, a strain previously shown to exert anti-obesity effects in preclinical studies [114,115]. This trial involved 100 overweight participants who were randomly assigned to either the experimental group (LMT1-48 for 12 weeks) or the placebo group. The results revealed significant reductions in body weight and abdominal adiposity, with no safety concerns [116].

A similar double-blind RCT, involving 106 adults aged 19–70 years with a BMI between 25 and 30 kg/m^2^, also showed that 12 weeks of LMT1-48 supplementation led to significant reductions in body fat mass and percentage, as well as enhanced lean mass, without significant adverse effects [117]. These promising results highlight the potential of LMT1-48 as an adjunctive therapy for overweight patients. However, further research is needed in pediatric populations.

An 8-week RCT examined the effects of two specific *Bifidobacterium* strains (*B. breve* BR03 and *B. breve* B632) in children with obesity and IR [118]. This probiotic intervention significantly improved insulin sensitivity, both at fasting and post-oral glucose tolerance test (OGTT), reduced Homeostatic Model Assessment of Insulin Resistance (HOMA-IR), and decreased waist circumference (WC), supporting weight loss [118]. These findings align with the growing body of literature on the beneficial effects of *Bifidobacterium* supplementation in both murine models and adult populations, particularly regarding weight loss and obesity-related comorbidities [119,120,121,122,123].

In contrast, a 6-month RCT found that multi-strain probiotic supplementation offered no significant advantage over lifestyle modification alone in improving obesity-related metabolic imbalances in children [124]. However, multi-strain probiotics such as *L. salivarius* AP-32, *L. rhamnosus* bv-77, and *B. animalis* CP-9 have been shown to reshape obesity-related gut dysbiosis and significantly improve lipid metabolism, leading to reductions in BMI [125].

A recent meta-analysis by Li et al. evaluated the effects of probiotics on various metabolic markers in children with obesity, including BMI, total cholesterol (TC), triglycerides (TG), high-density lipoprotein cholesterol (HDL-C), low-density lipoprotein cholesterol (LDL-C), adiponectin, leptin, and TNF-α [126]. Probiotics were found to improve HDL-C, LDL-C, adiponectin, and leptin levels, while significantly reducing TNF-α. However, no significant effects were observed on BMI, TC, or TG, likely due to limited sample sizes and few trials focused on children with overweight or obesity [126].

**Table 1:** Principal evidence on the role of probiotics in pediatric obesity.

References	Study Design	Population	Main Findings
Solito et al. [118]	Randomized, double-blind, placebo-controlled cross-over trial.	101 children and adolescents with obesity (mean age 12 years) with IR (defined by HOMA-IR > 2.5 or fasting insulin > 15 μU/mL) were randomly assigned to receive either a probiotic mix of *Bifidobacterium breve* BR03 and B632 (2 × 10^9^ CFU/day) or placebo for 8 weeks, followed by a 4-week washout and crossover. All subjects followed an isocaloric Mediterranean diet and lifestyle recommendations during the trial.	In the first 8-week phase, probiotics improved insulin sensitivity. Significant reductions were also observed in fasting insulin, WC, ALT, and fecal *E. coli* levels (all *p* < 0.05). SCFAs profiles remained stable in the probiotic arm, while placebo subjects exhibited increased acetic acid levels.
Rodrigo et al. [124]	Randomized, double-blind, placebo-controlled cross-over trial.	84 children with obesity and diagnosed with ultrasound-detected NAFLD/NASH were divided into probiotic group (*n* = 43) and into placebo group (*n* = 41). Participants in the probiotic group were randomized to receive a multi-strain probiotic supplement for 6 months. Both groups followed structured dietary and lifestyle modifications.	Both groups showed improvements in triglycerides, AST, ALT, AST/ALT ratio, and ALP levels, but only the placebo group reached statistical significance (all *p* < 0.05). Although BMI decreased significantly in the probiotic group, no significant difference was found compared to placebo (*p* > 0.05). Liver steatosis grade in USS improved from stage II–III to stage I in a small non-significant subset of probiotic-treated patients. Transient elastography showed no significant fibrosis improvement in either group (*p* > 0.05).
Chen et al. [125]	Randomized, double-blind, placebo-controlled trial.	82 children aged 6–18 with overweight or obesity were enrolled, and 53 participants (probiotic group, *n* = 27; placebo group, *n* = 26) completed the trial. Probiotic group received a multi-strain probiotic supplement (containing *Lactobacillus salivarius* AP-32, *L. rhamnosus* bv-77, and *Bifidobacterium animalis* CP-9) or placebo, along with standard dietary and exercise guidance.	After 12 weeks, the probiotic group showed increased serum HDL and adiponectin levels, while BMI, TC, LDL, leptin, and TNF-α levels decreased. Higher abundances of *B. animalis* and *Lactobacillus* spp. were associated with improved HDL (*p* = 0.029). *Lactobacillus* spp. levels were inversely correlated with lipid metabolism (*p* = 0.026), which in turn was positively associated with TC (*p* < 0.001) and LDL (*p* = 0.027).
Li et al. [126]	Systematic review and meta-analysis.	A systematic review through PubMed, Web of Science, Embase, Cochrane Library, SinoMed and CNKI was performed. 206 children with overweight or obesity from 4 randomized controlled trials were included. All participants underwent probiotic strains treatment with various treatment durations and geographic settings.	Compared to placebo group, probiotics increased HDL-C (*p* = 0.0001) and adiponectin levels (*p* < 0.0001), while reducing LDL-C (*p* = 0.04), leptin (*p* < 0.0001), and TNF-α (*p* < 0.0001). No significant effects were observed for TC or TG between the two groups (all *p* > 0.05). BMI changes were significant in the placebo group (*p* = 0.04).

Abbreviations: ALP: Alkaline Phosphatase; ALT: Alanine Aminotransferase; AST: Aspartate Transaminase; BMI: Body Mass Index; HDL: High-Density Lipoprotein; HOMA-IR: Homeostatic Model Assessment for Insulin Resistance; IR: Insulin Resistance; LDL: Low-Density Lipoprotein; NAFLD: Non-Alcoholic Fatty Liver Disease; NASH: Nonalcoholic Steatohepatitis; SCFAs: Short-Chain Fatty Acids; TC: Total Cholesterol; TG: Triglycerides; TNF-α: Tumor Necrosis Factor-alpha; USS: Ultrasound Scan; WC: waist circumference.

Overall, probiotic supplementation holds promise for modulating GM and supporting metabolic health in pediatric obesity. However, more research is required to clarify its mechanisms and confirm sustained benefits.

Future studies should also determine the most effective strains, suitable delivery methods, and appropriate dosages for children.

### 5.3. Prebiotics: An Adjunctive Viable Therapeutic Option

Prebiotics, defined as substrates selectively utilized by host microorganisms to confer health benefits [127], have also been explored as potential adjuvant therapies for pediatric obesity, with mixed results [102,128,129,130,131,132] (Table 2).

A recent review highlights that prebiotic supplementation can modulate enteroendocrine function and support healthy gut function, which subsequently influences lipid and glucose homeostasis and regulates appetite [133].

Nicolucci et al. examined the impact of oligofructose-enriched inulin in a cohort of children with overweight or obesity and found significant reductions in body weight z-score, percentage of body fat, and trunk fat percentage following 16 weeks of supplementation, compared to the placebo group [134]. Additionally, they observed a significant decrease in IL-6 levels and serum triglycerides in the intervention group, although changes in the serum inflammatory profile were not statistically significant [134].

A further 16-week RCT investigating the effects of oligofructose-enriched inulin on appetite control and energy intake in children with a BMI ≥ 85th percentile revealed increased fasting adiponectin and ghrelin levels, alongside significant improvements in subjective appetite ratings [135]. Furthermore, energy intake during a breakfast buffet was markedly reduced, particularly among older children in the intervention group [135].

Visuthranukul et al. assessed the relationships between inflammatory cytokines, adiposity and IR in children with obesity [136]. BMI z-score, fat mass index (FMI), trunk FMI, percent body fat, IL-1 and TNF-α all significantly decreased after the inulin intervention in all groups, but no significant differences were observed between groups [136].

At baseline, IL-6 correlated with fat mass and IR, supporting its role as a marker of systemic inflammation in pediatric obesity [136]. However, unlike to previous evidence, IL-6 levels increased during the study, likely due to increased physical activity across all groups [137,138,139,140].

Another RCT showed that inulin supplementation improved gut bacterial diversity and corrected microbiota dysbiosis, leading to the reversal of metabolic and clinical abnormalities in children with obesity [141]. The intervention increased fat-free mass and boosted the abundance of *Bifidobacterium*, along with several SCFA-producing bacteria, specific to the inulin group [141]. These results are in line with in vitro findings by Holmes et al., which showed that various over-the-counter (OTC) prebiotics enhanced SCFA production in the fecal microbiota of children [142]. However, the level of SCFA production varied depending on the prebiotic type and in-dividual microbiota composition [142].

**Table 2:** Principal evidence on the role of prebiotics in pediatric obesity.

References	Study Design	Population	Main Findings
Nicolucci et al. [134]	Randomized, double-blind, placebo-controlled trial	42 children aged 7 to 12 years with simple overweight or obesity were randomly assigned to receive either OI (8 g/day; *n* = 22) or an isocaloric maltodextrin placebo (*n* = 20) for 16 weeks.	Children who received prebiotic supplementation with OI experienced a 3.1% reduction in body weight z-score, a 2.4% decrease in total BFP, and a 3.8% decline in trunk fat. The OI group showed a 19% reduction in serum triglycerides and a 15% decrease in IL-6, whereas IL-6 increased by 25% in the placebo group. Microbiota analysis revealed a selective expansion of *Bifidobacterium* spp. and a reduction of *Bacteroides vulgatus* in the prebiotic group.
Hume et al. [135]	Randomized, double-blind, placebo-controlled trial	42 children aged 7 to 12 years with overweight or obesity were enrolled, and 38 participants concluded the 16-week trial. Prebiotic group (*n* = 20) received an 8 g/day of OI. Placebo group (*n* = 18) received an isocaloric maltodextrin. Appetite control was assessed both objectively (via energy intake) and subjectively (through visual analogue scales and parent-completed questionnaires).	Prebiotics significantly increased post-breakfast feelings of fullness (*p* = 0.04) and reduced the desire for further food intake compared to placebo (*p* = 0.03). Significant reduction in energy intake at the final breakfast buffet in children aged 11–12 years) compared to younger participants was found (*p* = 0.04). Fasting adiponectin and ghrelin levels significantly increased in the prebiotic group compared to placebo group (*p* = 0.04 and *p* = 0.03, respectively).
Visuthranukul et al. [136]	Randomized double-blind placebo-controlled trial	155 Thai children with obesity aged 7–15 years were divided in 3 groups: (i) children receiving inulin supplementation, (ii) children receiving maltodextrin placebo, and (iii) patients receiving dietary fiber counseling only.	Both intervention groups showed a significant reduction in BMI z-score, FMI, percent body fat, and trunk FMI (all *p* < 0.05). Both groups also demonstrated significant reductions in IL-1β (−34.8%) (*p* < 0.0001) and TNF-α levels (−25.8%) (*p* < 0.0001), while IL-6 increased (+21.5%, *p* = 0.006). IL-6 showed a positive correlation with percent body fat (r = 0.29, *p* = 0.008) and FMI (r = 0.25, *p* = 0.049). No differences between groups for cytokines or fecal calprotectin were detected.
Visuthranukul et al. [141]	Randomized, double-blind, placebo-controlled trial	143 Thai children aged 7 to 15 years with obesity randomly allocated to three arms: (i) intervention group receiving inulin, (ii) placebo group receiving isocaloric maltodextrin, and (iii) control group receiving only dietary fiber advice. All participants underwent standardized monthly follow-up for 6 months, with identical lifestyle counseling.	The intervention group showed a significant increase in microbial alpha-diversity (*p* < 0.05). This group also showed significant enrichment in beneficial taxa, particularly *Bifidobacterium*, *Blautia*, *Megasphaera*, and butyrate-producing bacteria such as *Agathobacter*, *Eubacterium coprostanoligenes*, and *Subdoligranulum* with associated clinical and metabolic improvements (all *p* < 0.05).

Abbreviations: BMI: Body Mass Index; BFP: Body Fat Percentage; FMI: Fat Mass Index; IL-1β: interleukin-1β; IL-6: Interleukin-6; OI: Oligofructose-enriched inulin; TNF-α: Tumor Necrosis Factor-alpha.

Although promising, evidence supporting the benefits of prebiotic supplementation in children whit overweight or obesity remains limited [131,132,133].

Further research is needed to clarify its interactions with the host microbiota and to understand the long-term effects of these interventions.

### 5.4. Synbiotics: A Synergistic Strategy

Synbiotics are defined as mixtures of live microorganisms and substrates selectively utilized by host microorganisms, which together confer health benefits to the host [143]. In this context, “host microorganisms” include both autochthonous (resident or naturally colonizing) and allochthonous (externally applied, such as probiotics) microorganisms, each of which may be targeted by the substrates present in the synbiotic formulation [143].

Growing interest surrounds the use of synbiotics for various chronic diseases in adults [144,145,146,147], and increasingly, in pediatric obesity [109,148,149,150] (Table 3).

Indeed, the co-administration of prebiotics in synbiotics has been found to support the colonization and metabolic activity of probiotic strains, thereby enhancing their beneficial effects [149,151].

An open-label RCT including children with primary obesity demonstrated that synbiotic supplementation significantly improved anthropometric measures, as well as serum TC, LDL-C, and total oxidative stress levels [149]. Similarly, another RCT conducted on children and adolescents with obesity showed a significant reduction in waist-to-height ratio (WHtR) in the group receiving a synbiotic containing *L. coagulans* SC-208, *L. indicus* HU36, and fructooligosaccharides (FOS), compared to baseline [152]. However, no significant changes were observed in other anthropometric measures or body composition between the groups [152].

Despite these promising results, current evidence regarding the use of synbiotics in pediatric obesity remains limited [149,152].

Further well-designed clinical trials are needed to better evaluate the efficacy, safety, and long-term effects of synbiotic interventions in children with obesity.

### 5.5. Postbiotics: An Emerging Therapeutic Target

Postbiotics—defined as inanimate microorganisms or their components that confer a health benefit on the host [101,153]—have recently gained increasing attention as potential modulators of metabolic health [153,154].

Recent studies have highlighted their ability to influence host energy balance, immune responses, and systemic inflammation through bioactive molecules such as SCFAs, exopolysaccharides, lipoteichoic acid, and peptidoglycan-derived components [154,155].

In adults, postbiotics have been associated with improvements in insulin sensitivity, lipid metabolism, and low-grade inflammation, suggesting potential utility in obesity and related metabolic disorders [156]. A recent RCT demonstrated that pasteurized *A. muciniphila* improved insulin sensitivity and reduced insulinemia in overweight adults [157]. Another study highlighted the ability of muramyl dipeptide (MDP)—a bacterially derived postbiotic—to enhance insulin sensitivity and reduce adipose inflammation via Nucleotide-binding Oligomerization Domain-containing protein 2 (NOD2) signaling in in vivo models [158].

Moreover, SCFAs, bacterial cell wall components, and bioactive peptides have been shown to modulate metabolic hormones (e.g., GLP-1, PYY), activate AMPK, and attenuate inflammatory signaling [159].

However, evidence in pediatric populations remains scarce [109,156]. Luzzi et al. evaluated the effects of prebiotics, probiotics, synbiotics, and postbiotics in adolescents with metabolic syndrome [109]. Preliminary results suggested that postbiotic supplementation improved insulin sensitivity, lipid profiles, and low-grade inflammation. However, the limited sample size and short follow-up duration of the study highlight the need for larger, randomized pediatric trials to better establish the therapeutic potential of postbiotics in the complex management of pediatric obesity [109].

Despite these limitations, postbiotics may represent a safe and feasible adjunct to conventional interventions, with the potential to modulate low-grade inflammation, gut barrier integrity, and energy metabolism. Future proof-of-concept studies are war-ranted to clarify their role in pediatric obesity and to establish standardized formulations, dosages, and treatment durations.

### 5.6. FMT: A Novel Insight into Future Therapeutic Approaches

FMT is a therapeutic intervention involving the transfer of stool from a healthy donor to the gastrointestinal tract of a recipient aimed at restoring microbial diversity, reducing intestinal permeability, and suppressing the pro-inflammatory pathways associated with dysbiosis [160,161,162].

**Figure 3:**
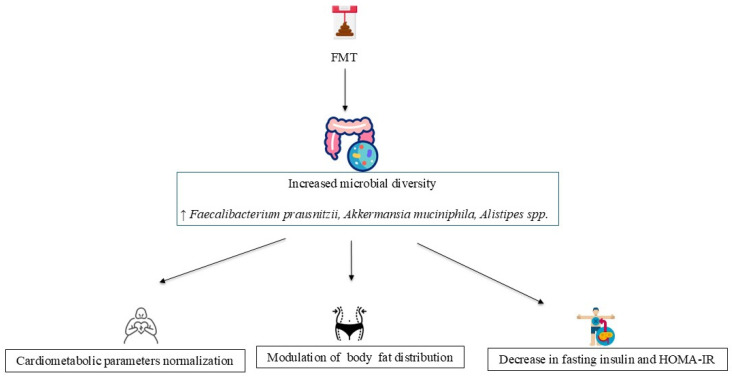
Therapeutic insights from FMT in pediatric obesity. Abbreviations: HOMA-IR: Homeostatic Model Assessment for Insulin Resistance; FMT: Fecal Microbiota Transplant.

Given its potential to modulate key pathophysiological mechanisms, FMT has gained considerable attention as a potential treatment for metabolic disorders, including obesity [161,162,163] (Figure 3).

Preclinical studies in murine models have shown that transferring microbiota from obese donors to germ-free mice induces adiposity and metabolic disturbances, while the transfer from lean donors improves insulin sensitivity and promotes a metabolically healthier phenotype [63,164].

In human trials, several adult studies have reported transient improvements in metabolic profiles, such as insulin sensitivity, inflammatory markers, and GM composition, following FMT from lean donors in individuals with metabolic syndrome. However, these benefits have not consistently translated into significant reductions in body weight, underscoring the importance of concurrent lifestyle interventions—such as adherence to the MD and physical activity—in managing obesity [165,166,167].

Evidence on FMT in pediatric populations remains limited [168,169,170]. The Gut Bugs Trial by Leong et al. is one of the most notable studies, in which adolescents with obesity received a single course of oral encapsulated FMT from lean donors or a placebo, with 26 weeks of follow-up [168].

While no significant difference in BMI z-scores was observed, FMT led to favorable changes in body fat distribution, particularly reductions in visceral adiposity, as well as short-term improvements in IR. In participants with undiagnosed metabolic syndrome, FMT was associated with a greater likelihood of metabolic normalization compared to placebo [168].

Microbiome analysis revealed increased diversity and higher levels of beneficial taxa, including *F. prausnitzii*, *A. muciniphila*, and *Alistipes* spp., which are known to exert anti-inflammatory effects and regulate metabolic functions [168,171,172].

Despite these promising findings, the overall evidence for pediatric FMT remains sparse [168,169,170,173] (Table 3).

**Table 3:** Principal evidence on the role of Synbiotics and FMT in pediatric obesity.

References	Study Design	Population	Main Findings
Ipar et al. [149]	Randomized controlled trial	86 children and adolescents with primary obesity were enrolled and 77 children (aged 5–17 years) completed the one- month intervention. Participants were randomly divided in two groups: (i) patients (*n* = 35) receiving standard lifestyle modifications including reduced caloric intake and increased physical activity; (ii) patients (*n* = 42) receiving the same lifestyle advice in addition to a daily synbiotic supplement for a 30-day period. 40 children were enrolled as a control group.	Children receiving synbiotic supplementation showed a significantly greater reduction in both body weight (*p* < 0.001) and BMI (*p* < 0.001) compared to those undergoing standard intervention alone. Significant improvements in anthropometric measures were also reported in the synbiotic group (all *p* < 0.05). Oxidative stress and TC and LDL levels significantly decreased in the synbiotic group (all *p* < 0.05).
Atazadegan et al. [152]	Randomized, double-blind, placebo-controlled trial	60 children and adolescents overweight or with obesity aged 8–18 years were randomly allocated in two group: (i) patients receiving a synbiotic supplement-comprising *Lactobacillus coagulans* SC-208 and *Lactobacillus indicus* HU36 (each at 6 × 10^9^ CFU) with FOS; (ii) patients receiving placebo for 8 weeks.	WHtR significantly decreased in the synbiotic group compared to baseline (*p* = 0.05). No significant differences for other anthropometric indices were found compared to placebo (all *p* > 0.05).
Leong et al. [168]	Randomized, double-blind, placebo-controlled trial	87 New Zealand adolescents aged 14–18 years with obesity stratified by sex and randomly assigned 1:1 to receive either a single course of encapsulated FMT derived from healthy lean same-sex donors (*n* = 42) or placebo (*n* = 45). The intervention was followed by a 26-week monitoring period. Baseline metabolic syndrome was assessed in a subset of participants.	Participants of the FMT group experienced a significant and sustained reduction in the A/G fat ratio at 6, 12, and 26 weeks compared to placebo (all *p* < 0.05). No significant effects were found on insulin sensitivity, liver enzymes, lipid profile, inflammatory markers, total body fat percentage, or quality of life (all *p* > 0.05). In children with metabolic syndrome at baseline, FMT was associated with a significantly higher resolution rate of the condition by week 26 (from 18 to 4 cases) compared to placebo (from 13 to 10 cases), with an aOR of 0.06 (95% CI 0.01–0.45; *p* = 0.007).
Fahim et al. [170]	Systematic review and meta-analysis	17 RCT examining data of 838 children and adolescents aged 0 to 19 years with overweight or obesity across multiple countries were included. Targeted interventions included probiotics, prebiotics, synbiotics, SCFAs, and fecal microbiota transplantation FMT.	In adolescents aged 10–19 years, probiotics and FMT did not show significant effects on anthropometric or cardiometabolic parameters. In patients aged 0–19 years, prebiotics were associated with modest but statistically significant reductions in BMI (MD −0.70, 95% CI −1.25 to −0.15) and body weight (MD −1.5 kg, 95% CI −2.61 to −0.39) compared to placebo. Synbiotics showed reduced SBP in one study (*n* = 56). SCFAs reduced WC (MD −5.08 cm, 95% CI −7.40 to −2.76) and BMI (MD −2.26, 95% CI −3.24 to −1.28). Very low overall certainty due to methodological limitations, small samples, and sparse outcome reporting.
Wilson et al. [173]	Randomized, double-blind, placebo-controlled trial	87 Australian adolescents with obesity aged 14–18 years randomly allocated to receive either encapsulated FMT from healthy donors (*n* = 42) or placebo capsules (*n* = 45). After the 26-week double-blind phase, 55 participants (27 FMT, 28 placebo) were followed up at 4 years. Baseline metabolic syndrome status enabled stratified analyses.	After 4 years, adjusted analyses showed no significant effect of FMT on BMI compared to placebo. Adolescents who received FMT exhibited reduced WC (−10.0 cm, *p* = 0.026), total body fat percentage (−4.8%, *p* = 0.024), and hs-CRP levels (−68%, *p* = 0.002) and a lower metabolic syndrome severity score (−0.58, *p* = 0.003). HDL cholesterol levels increased modestly in the FMT group (*p* = 0.037).

Abbreviations: A/G: Android-to-Gynoid; aOR: Adjusted Odds Ratio; BMI: Body Mass Index; CFU: Colony Forming Unit; FMT: Fecal Microbiota Transplantation; FOS: Fructooligosaccharides; LDL: Low-Density Lipoprotein; MD: Mean Difference; SBP: Systolic Blood Pressure; SCFAs: Short-Chain Fatty Acids; TC: Total Cholesterol; WC: waist circumference; WHtR: Waist-To-Height Ratio.

A recent Cochrane systematic review examining GM-based interventions for obesity in individuals up to 19 years of age found that FMT likely yields minimal or no differences in key obesity-related outcomes, such as BMI, WC, body fat percentage, and blood pressure when compared to placebo [170]. Similar studies in pediatric patients with *Clostridioides difficile* infections and ulcerative colitis (UC) have also failed to demonstrate clinically meaningful changes in BMI or adiposity [174,175].

While current data do not support routine FMT use for pediatric obesity, improvements in visceral adiposity and resolution of metabolic syndrome in select individuals suggest that FMT may offer therapeutic potential for certain subsets of children with obesity [167,168,169].

However, the evidence remains limited due to methodological constraints, such as small sample sizes, short follow-up periods, and a lack of RCTs specifically targeting pediatric obesity.

Future studies are necessary to identify the microbial taxa responsible for the observed cardiometabolic benefits and to develop standardized microbial mixtures for targeted interventions in pediatric obesity and related metabolic disorders [169,172,174].

### 5.7. Translational Insights from Murine Models

Growing evidence from murine models has reinforced the pathogenic role of GM composition in the development of obesity-related metabolic impairments, providing a rationale for microbiota-targeted therapies in pediatric populations with obesity [176,177].

Experimental data have consistently demonstrated that diet-induced obesity models, where mice are colonized with microbiota from obese donors, develop increased fat accumulation, impaired insulin sensitivity, and intestinal barrier dysfunction. In contrast, transplantation of microbiota from lean or metabolically healthy donors confers resistance to lipid accumulation, enhances glucose tolerance, and strengthens tight junction integrity, even in the presence of a high-fat diet [176,178,179].

A recent murine study showed that FMT from donors fed a methionine-restricted diet—a regimen known to improve insulin sensitivity, reduce fat mass, and promote metabolic health—resulted in significant reductions in adiposity and improved metabolic markers in recipient mice, despite no dietary changes in the recipient animals [180]. This underscores the importance of donor nutritional status in determining the functional properties and therapeutic potential of transferred microbiota [180].

A recent insightful study exploring fecal virome transplantation (FVT) demonstrated the selected removal of eukaryotic viruses while preserving bacteriophage [181].

Mice receiving FVT exhibited improved glycemic control and reduced adipose tissue inflammation, suggesting a potential role for non-bacterial components of the gut microbiota—particularly bacteriophages—in metabolic regulation. These findings open new avenues for future research into the therapeutic potential of bacteriophages in metabolic health [181].

In another study, FMT was performed by transferring microbiota from children with obesity into germ-free mice [176]. Although the recipients exhibited persistent alterations in metabolite profiles—such as increased levels of indole-3-acetic acid and methyllysine—no significant differences were observed in body weight, food intake, or glucose metabolism compared to mice colonized with microbiota from lean donors.

These findings suggest that, while microbial composition can influence certain metabolic pathways, FMT alone may be insufficient to drive overt phenotypic changes in metabolism. This highlights the complex, multifactorial nature of obesity and points to the need for combinatorial therapeutic strategies—particularly the integration of dietary modifications and lifestyle interventions—to enhance the efficacy of microbiota-targeted therapies in pediatric obesity [176].

Despite certain limitations and variability across studies, common pathophysiological mechanisms linking GM and metabolic regulation continue to emerge. Beneficial metabolic effects following FMT have been associated with the enrichment of specific microbial taxa such as *Akkermansia*, *Odoribacter*, and certain groups within *Clostridiales* [178,182]. These taxa are recognized for their anti-inflammatory properties and insulin-sensitizing effects, suggesting a potential mechanistic link between micro-bial composition and improved metabolic outcomes [172,178,182].

Nevertheless, the effectiveness of FMT in modulating adiposity and glucose metabolism appears to be influenced by multiple interacting factors, including donor microbiota composition, recipient dietary habits, and the intrinsic resilience of the host microbiome [176,180,183].

Further experimental evidence indicates that younger recipient age and antibiotic-induced microbial depletion enhance donor microbiota engraftment and improve metabolic outcomes, underscoring early life as a critical therapeutic window for microbiota-targeted interventions [184,185].

Collectively, these preclinical findings provide robust support for the biological plausibility of microbiota-modulating strategies in pediatric obesity [176,180,181,185,186].

However, the translation of these encouraging results from murine models to clinical practice necessitates rigorously designed human trials—particularly involving children and adolescents—to evaluate the safety, efficacy, and long-term impact of micro-biota-based therapies in this vulnerable population.

## 6. Limitations

While the GM has emerged as an insightful target in the prevention and treatment of pediatric obesity, current evidence supporting its therapeutic manipulation remains preliminary and is subject to several important limitations [113,164,170]. A significant proportion of the available data is derived from preclinical models or adult cohorts, with relatively few longitudinal, large-scale, and randomized controlled trials specifically focused on pediatric populations. Additionally, considerable heterogeneity in study methodologies—including variation in microbiome profiling techniques, dietary assessments, intervention types, and clinical outcome measures—hampers the comparability and reproducibility of findings. Moreover, the GM exhibits high interindividual variability, influenced by host genetics, age, developmental stage, diet, and environmental exposures, which complicates the identification of universal microbial targets [66,187]. Notably, while microbial associations with obesity-related phenotypes are well established, causality remains difficult to determine due to the complex bidirectional interactions between host and microbiota.

Emerging therapies such as FMT and engineered probiotics, although promising, currently lack sufficient long-term safety and efficacy data in pediatric settings [170,184].

Thus, while microbiota-directed interventions offer novel avenues for research and potential clinical application, they should currently be approached with caution and considered complementary to, rather than replacements for, established evidence-based strategies in pediatric obesity management.

## 7. Conclusions

Targeting the GM represents a promising, yet still evolving, strategy for the management of pediatric obesity. Although microbial dysbiosis has been consistently linked to metabolic dysfunction—via mechanisms including increased energy harvest, low-grade inflammation, and disruption of endocrine signaling—the translation of these insights into effective clinical interventions remains challenging.

Therapeutic approaches such as prebiotics, probiotics, synbiotics, and dietary modification have shown potential in restoring microbial homeostasis and improving metabolic outcomes. However, their efficacy and long-term impact in pediatric populations require further validation through well-designed, large-scale clinical studies.

More advanced approaches, such as FMT and engineered microbial consortia, offer intriguing possibilities but raise concerns regarding long-term safety, standardization, and ethical considerations, particularly in children. In parallel, recent advances in metagenomics and systems biology are facilitating the development of microbiota-informed, precision-based therapeutic strategies. Notably, microbial metabolites—including short-chain fatty acids, secondary bile acids, and indole derivatives—have emerged as key mediators of host energy regulation, appetite control, and immune modulation.

Nevertheless, significant challenges remain. Interindividual variability, age-dependent microbiota dynamics, environmental exposures, and host genetic factors complicate the design of universally effective interventions. Moreover, many of the proposed therapies lack robust longitudinal data in pediatric cohorts, limiting their current translational applicability.

Thus, microbiota-targeted interventions should be viewed not as standalone treatments but as complementary components within a multifaceted framework for obesity management. Future research should prioritize the development of safe, effective, and scalable strategies that integrate microbiota modulation with established dietary, behavioral, and clinical approaches. A comprehensive understanding of GM–host interactions—particularly during critical developmental windows—will be essential to inform targeted, sustainable interventions for the prevention and treatment of pediatric obesity.

## 8. Future Directions

Given the rapidly evolving landscape of GM-targeted strategies, future research should prioritize elucidating the intricate and dynamic interactions between the GM and host metabolic, immune, and neuroendocrine systems during early life.

Particular emphasis should be placed on identifying specific microbial taxa, metabolic pathways, and bioactive metabolites that either promote or mitigate the development of obesity.

To fill this gap, large-scale, longitudinal cohort studies and rigorously designed RCTs are essential to assess the efficacy, safety, and long-term impact of gut microbiota-modulating interventions—including probiotics, prebiotics, synbiotics, postbiotics, and tailored dietary approaches.

The integration of multi-omics technologies—such as metagenomics, metabolomics, and transcriptomics —alongside advanced computational modeling, will be pivotal in characterizing individual microbiome signatures and informing personalized therapeutic strategies.

Moreover, a comprehensive understanding of environmental, behavioral, and socioeconomic determinants of GM composition will be critical to enhance the translational value and scalability of these interventions.

Advancing microbiome-informed approaches to the early prevention and effective management of pediatric obesity will require a multidisciplinary framework that bridges basic science, clinical research, and public health.

## Data Availability

The original contributions presented in the paper are included in the review article. Further inquiries can be directed to the corresponding author.

## Abbreviations

The following abbreviations are used in this manuscript:

FMT	fecal microbiota transplantation
GLP-1	Glucagon-like peptide-1
GM	Gut microbiota
HDL-C	high-density lipoprotein cholesterol
IR	Insulin resistance
LDL-C	low-density lipoprotein cholesterol
MASLD	Metabolic Dysfunction-Associated Steatotic Liver Disease
MD	Mediterranean diet
NAFLD	Non-alcoholic fatty liver disease
RCT	randomized controlled trial
SCFAs	Short-Chain Fatty Acids
TC	total cholesterol
T2D	type 2 diabetes
TG	triglycerides
TLR4	Toll-like receptor 4

## References

[1] Hannon T.S., Arslanian S.A. (2023). Obesity in Adolescents. N. Engl. J. Med..

[2] The Lancet Diabetes & Endocrinology (2022). Childhood Obesity: A Growing Pandemic. Lancet Diabetes Endocrinol..

[3] Kerr J.A., Patton G.C., Cini K.I., Abate Y.H., Abbas N., Abd Al Magied A.H.A., Abd ElHafeez S., Abd-Elsalam S., Abdollahi A., Abdoun M. (2025). Global, Regional, and National Prevalence of Child and Adolescent Overweight and Obesity, 1990–2021, with Forecasts to 2050: A Forecasting Study for the Global Burden of Disease Study 2021. Lancet.

[4] Spinelli A., Buoncristiano M., Kovacs V.A., Yngve A., Spiroski I., Obreja G., Starc G., Pérez N., Rito A.I., Kunešová M. (2019). Prevalence of Severe Obesity among Primary School Children in 21 European Countries. Obes. Facts.

[5] Abarca-Gómez L., Abdeen Z.A., Hamid Z.A., Abu-Rmeileh N.M., Acosta-Cazares B., Acuin C., Adams R.J., Aekplakorn W., Afsana K., Aguilar-Salinas C.A. (2017). Worldwide Trends in Body-Mass Index, Underweight, Overweight, and Obesity from 1975 to 2016: A Pooled Analysis of 2416 Population-Based Measurement Studies in 128 9 Million Children, Adolescents, and Adults. Lancet.

[6] Zhang X., Liu J., Ni Y., Yi C., Fang Y., Ning Q., Shen B., Zhang K., Liu Y., Yang L. (2024). Global Prevalence of Overweight and Obesity in Children and Adolescents: A Systematic Review and Meta-Analysis. JAMA Pediatr..

[7] Zhu H., Yi X., He M., Wu S., Li M., Gao S. (2025). Exploring the Interplay of Genetic Variants and Environmental Factors in Childhood Obesity: A Systematic Review and Meta-Analysis. Metabolism.

[8] Ernest D.K., Onugha E.A., Singh B., Sharma S.V., Dave J.M. (2025). A Scoping Review of the Social Determinants of Pediatric and Adolescent Obesity. Int. J. Pediatr..

[9] Faienza M.F., Baima J., Cecere V., Monteduro M., Farella I., Vitale R., Antoniotti V., Urbano F., Tini S., Lenzi F.R. (2025). Fructose Intake and Unhealthy Eating Habits Are Associated with MASLD in Pediatric Obesity: A Cross-Sectional Pilot Study. Nutrients.

[10] Sun M., Sun H. (2025). Recent Prevalence and Trends of Obesity and Metabolic Dysfunction-associated Steatotic Liver Disease (MASLD) among US Adolescents: 1999 to 2020. Pediatr. Obes..

[11] Forcina G., Luciano M., Frattolillo V., Mori S., Monaco N., Guarino S., Marzuillo P., Miraglia Del Giudice E., Di Sessa A. (2024). Kidney Damage in Pediatric Obesity: Insights from an Emerging Perspective. JCM.

[12] Chung G.E., Yu S.J., Yoo J.-J., Cho Y., Lee K., Shin D.W., Kim Y.J., Yoon J.-H., Han K., Cho E.J. (2025). Metabolic Dysfunction-Associated Steatotic Liver Disease Increases Cardiovascular Disease Risk in Young Adults. Sci. Rep..

[13] Obrycki Ł., Skoczyński K., Sikorski M., Koziej J., Mitoraj K., Pilip J., Pac M., Feber J., Litwin M. (2025). Current Etiology of Hypertension in European Children—Factors Associated with Primary Hypertension. Pediatr. Nephrol..

[14] Badr M., El-Rabaa G., Freiha M., Kędzia A., Niechciał E. (2025). Endocrine Consequences of Childhood Obesity: A Narrative Review. Front. Endocrinol..

[15] Maffeis C., Olivieri F., Valerio G., Verduci E., Licenziati M.R., Calcaterra V., Pelizzo G., Salerno M., Staiano A., Bernasconi S. (2023). The Treatment of Obesity in Children and Adolescents: Consensus Position Statement of the Italian Society of Pediatric Endocrinology and Diabetology, Italian Society of Pediatrics and Italian Society of Pediatric Surgery. Ital. J. Pediatr..

[16] Seo Y.-G., Lim H., Kim Y., Ju Y.-S., Lee H.-J., Jang H.B., Park S.I., Park K.H. (2019). The Effect of a Multidisciplinary Lifestyle Intervention on Obesity Status, Body Composition, Physical Fitness, and Cardiometabolic Risk Markers in Children and Adolescents with Obesity. Nutrients.

[17] Ho M., Garnett S.P., Baur L., Burrows T., Stewart L., Neve M., Collins C. (2012). Effectiveness of Lifestyle Interventions in Child Obesity: Systematic Review with Meta-Analysis. Pediatrics.

[18] Manco M. (2025). Reframing Obesity in Children. JAMA Pediatr..

[19] Yanovski J.A., Krakoff J., Salaita C.G., McDuffie J.R., Kozlosky M., Sebring N.G., Reynolds J.C., Brady S.M., Calis K.A. (2011). Effects of Metformin on Body Weight and Body Composition in Obese Insulin-Resistant Children. Diabetes.

[20] Masarwa R., Brunetti V.C., Aloe S., Henderson M., Platt R.W., Filion K.B. (2021). Efficacy and Safety of Metformin for Obesity: A Systematic Review. Pediatrics.

[21] Evia-Viscarra M.L., Rodea-Montero E.R., Apolinar-Jiménez E., Muñoz-Noriega N., García-Morales L.M., Leaños-Pérez C., Figueroa-Barrón M., Sánchez-Fierros D., Reyes-García J.G. (2012). The Effects of Metformin on Inflammatory Mediators in Obese Adolescents with Insulin Resistance: Controlled Randomized Clinical Trial. J. Pediatr. Endocrinol. Metab..

[22] Weghuber D., Barrett T., Barrientos-Pérez M., Gies I., Hesse D., Jeppesen O.K., Kelly A.S., Mastrandrea L.D., Sørrig R., Arslanian S. (2022). Once-Weekly Semaglutide in Adolescents with Obesity. N. Engl. J. Med..

[23] Kelly A.S., Auerbach P., Barrientos-Perez M., Gies I., Hale P.M., Marcus C., Mastrandrea L.D., Prabhu N., Arslanian S. (2020). A Randomized, Controlled Trial of Liraglutide for Adolescents with Obesity. N. Engl. J. Med..

[24] Armstrong S.C., Bolling C.F., Michalsky M.P., Reichard K.W., Haemer M.A., Muth N.D., Rausch J.C., Rogers V.W., Heiss K.F., SECTION ON OBESITY, SECTION ON SURGERY (2019). Pediatric Metabolic and Bariatric Surgery: Evidence, Barriers, and Best Practices. Pediatrics.

[25] Pratt J.S.A., Browne A., Browne N.T., Bruzoni M., Cohen M., Desai A., Inge T., Linden B.C., Mattar S.G., Michalsky M. (2018). ASMBS Pediatric Metabolic and Bariatric Surgery Guidelines, 2018. Surg. Obes. Relat. Dis..

[26] Castaner O., Goday A., Park Y.-M., Lee S.-H., Magkos F., Shiow S.-A.T.E., Schröder H. (2018). The Gut Microbiome Profile in Obesity: A Systematic Review. Int. J. Endocrinol..

[27] Cho K.Y. (2023). Association of Gut Microbiota with Obesity in Children and Adolescents. Clin. Exp. Pediatr..

[28] Schoultz I., Claesson M.J., Dominguez-Bello M.G., Fåk Hållenius F., Konturek P., Korpela K., Laursen M.F., Penders J., Roager H., Vatanen T. (2025). Gut Microbiota Development across the Lifespan: Disease Links and Health-promoting Interventions. J. Intern. Med..

[29] Wang H.-X., Wang Y.-P. (2016). Gut Microbiota-Brain Axis. Chin. Med. J..

[30] Beam A., Clinger E., Hao L. (2021). Effect of Diet and Dietary Components on the Composition of the Gut Microbiota. Nutrients.

[31] Koller A.M., Săsăran M.O., Mărginean C.O. (2025). The Role of Gut Microbiota in Pediatric Obesity and Metabolic Disorders: Insights from a Comprehensive Review. Nutrients.

[32] Rondanelli M., Borromeo S., Cavioni A., Gasparri C., Gattone I., Genovese E., Lazzarotti A., Minonne L., Moroni A., Patelli Z. (2025). Therapeutic Strategies to Modulate Gut Microbial Health: Approaches for Chronic Metabolic Disorder Management. Metabolites.

[33] Abenavoli L., Scarpellini E., Colica C., Boccuto L., Salehi B., Sharifi-Rad J., Aiello V., Romano B., De Lorenzo A., Izzo A.A. (2019). Gut Microbiota and Obesity: A Role for Probiotics. Nutrients.

[34] Delzenne N.M., Neyrinck A.M., Bäckhed F., Cani P.D. (2011). Targeting Gut Microbiota in Obesity: Effects of Prebiotics and Probiotics. Nat. Rev. Endocrinol..

[35] Jones L., Kumar J., Mistry A., Sankar Chittoor Mana T., Perry G., Reddy V.P., Obrenovich M. (2019). The Transformative Possibilities of the Microbiota and Mycobiota for Health, Disease, Aging, and Technological Innovation. Biomedicines.

[36] Dominguez-Bello M.G., Blaser M.J., Ley R.E., Knight R. (2011). Development of the Human Gastrointestinal Microbiota and Insights from High-Throughput Sequencing. Gastroenterology.

[37] Ley D., Desseyn J.-L., Gouyer V., Plet S., Tims S., Renes I., Mischke M., Gottrand F. (2019). Early Life Nutrition Influences Susceptibility to Chronic Inflammatory Colitis in Later Life. Sci. Rep..

[38] U-Din M., Ahmed B.A., Syed S.A., Ong F.J., Oreskovich S.M., Gunn E., Surette M.G., Punthakee Z., Steinberg G.R., Morrison K.M. (2024). Characteristics of Abdominal Visceral Adipose Tissue, Metabolic Health and the Gut Microbiome in Adults. J. Clin. Endocrinol. Metab..

[39] Yarahmadi A., Afkhami H., Javadi A., Kashfi M. (2024). Understanding the Complex Function of Gut Microbiota: Its Impact on the Pathogenesis of Obesity and beyond: A Comprehensive Review. Diabetol. Metab. Syndr..

[40] Chen X., You L., Jia Y. (2025). The Role of Probiotics in Adolescents’ Obesity. Front. Cell. Infect. Microbiol..

[41] Faienza M.F., Urbano F., Anaclerio F., Moscogiuri L.A., Konstantinidou F., Stuppia L., Gatta V. (2024). Exploring Maternal Diet-Epigenetic-Gut Microbiome Crosstalk as an Intervention Strategy to Counter Early Obesity Programming. Curr. Issues Mol. Biol..

[42] Ortiz-Samur N.S., Vijaya A.K., Burokas A., Mela V. (2025). Exploring the Role of Microglial Cells in the Gut–Brain Axis Communication: A Systematic Review. J. Neurochem..

[43] Ben-Azu B., Del Re E.C., VanderZwaag J., Carrier M., Keshavan M., Khakpour M., Tremblay M.-È. (2023). Emerging Epigenetic Dynamics in Gut-Microglia Brain Axis: Experimental and Clinical Implications for Accelerated Brain Aging in Schizophrenia. Front. Cell. Neurosci..

[44] Martínez-Montoro J.I., Martín-Núñez G.M., González-Jiménez A., Garrido-Sánchez L., Moreno-Indias I., Tinahones F.J. (2024). Interactions between the Gut Microbiome and DNA Methylation Patterns in Blood and Visceral Adipose Tissue in Subjects with Different Metabolic Characteristics. J. Transl. Med..

[45] Nohesara S., Abdolmaleky H.M., Dickerson F., Pinto-Tomás A.A., Jeste D.V., Thiagalingam S. (2024). Maternal Gut Microbiome-Mediated Epigenetic Modifications in Cognitive Development and Impairments: A New Frontier for Therapeutic Innovation. Nutrients.

[46] Fasano A., Chassaing B., Haller D., Flores Ventura E., Carmen-Collado M., Pastor N., Koren O., Berni Canani R. (2024). Microbiota during Pregnancy and Early Life: Role in Maternal−neonatal Outcomes Based on Human Evidence. Gut Microbes.

[47] Li N., Liu H.-Y., Liu S.-M. (2024). Deciphering DNA Methylation in Gestational Diabetes Mellitus: Epigenetic Regulation and Potential Clinical Applications. Int. J. Mol. Sci..

[48] Woo V., Alenghat T. (2022). Epigenetic Regulation by Gut Microbiota. Gut Microbes.

[49] Shock T., Badang L., Ferguson B., Martinez-Guryn K. (2021). The Interplay between Diet, Gut Microbes, and Host Epigenetics in Health and Disease. J. Nutr. Biochem..

[50] Koleva P., Bridgman S., Kozyrskyj A. (2015). The Infant Gut Microbiome: Evidence for Obesity Risk and Dietary Intervention. Nutrients.

[51] Fan Y., Pedersen O. (2021). Gut Microbiota in Human Metabolic Health and Disease. Nat. Rev. Microbiol..

[52] Federici M. (2019). Gut Microbiome and Microbial Metabolites: A New System Affecting Metabolic Disorders. J. Endocrinol. Investig..

[53] Rezabakhsh A., Habtemariam S., Parvizi R., Meddahi-Pellé A., Ruiz V.R., Pavon-Djavid G., Barzgari A. (2025). The Gut-Heart Axis: A Correlation between Paneth Cells’ Dysfunction, Microbiome Dysbiosis, and Cardiovascular Diseases. Cell Commun. Signal..

[54] Turnbaugh P.J., Ley R.E., Hamady M., Fraser-Liggett C.M., Knight R., Gordon J.I. (2007). The Human Microbiome Project. Nature.

[55] Eckburg P.B., Bik E.M., Bernstein C.N., Purdom E., Dethlefsen L., Sargent M., Gill S.R., Nelson K.E., Relman D.A. (2005). Diversity of the Human Intestinal Microbial Flora. Science.

[56] Shabani M., Ghoshehy A., Mottaghi A.M., Chegini Z., Kerami A., Shariati A., Taati Moghadam M. (2025). The Relationship between Gut Microbiome and Human Diseases: Mechanisms, Predisposing Factors and Potential Intervention. Front. Cell. Infect. Microbiol..

[57] Carrizales-Sánchez A.K., García-Cayuela T., Hernández-Brenes C., Senés-Guerrero C. (2021). Gut Microbiota Associations with Metabolic Syndrome and Relevance of Its Study in Pediatric Subjects. Gut Microbes.

[58] De Moura E Dias M., Ribeiro M.G.C., Kravchychyn A.C.P., Hermsdorff H.H.M. (2025). Microbiota-Gut-Brain Axis and Impaired Satiety in Individuals with Obesity: A Potentially Bidirectional Association. Curr. Nutr. Rep..

[59] Mogoş G.F.R., Manciulea M., Enache R.-M., Pavelescu L.A., Popescu (Roşu) O.A., Cretoiu S.M., Marinescu I. (2025). Intestinal Microbiota in Early Life: Latest Findings Regarding the Role of Probiotics as a Treatment Approach for Dysbiosis. Nutrients.

[60] Cani P.D., Amar J., Iglesias M.A., Poggi M., Knauf C., Bastelica D., Neyrinck A.M., Fava F., Tuohy K.M., Chabo C. (2007). Metabolic Endotoxemia Initiates Obesity and Insulin Resistance. Diabetes.

[61] Liu L., Li M., Qin Y., Liu Y., Li M., Lian B., Guo R., Xiao Y., Yin C. (2025). Childhood Obesity and Insulin Resistance Is Correlated with Gut Microbiome Serum Protein: An Integrated Metagenomic and Proteomic Analysis. Sci. Rep..

[62] Iqbal F., Shenoy P.A., Lewis L.E.S., Siva N., Purkayastha J., Eshwara V.K. (2025). Influence of Perinatal Antibiotic on Neonatal Gut Microbiota: A Prospective Cohort Study. BMC Pediatr..

[63] Ridaura V.K., Faith J.J., Rey F.E., Cheng J., Duncan A.E., Kau A.L., Griffin N.W., Lombard V., Henrissat B., Bain J.R. (2013). Gut Microbiota from Twins Discordant for Obesity Modulate Metabolism in Mice. Science.

[64] Nohesara S., Mostafavi Abdolmaleky H., Pirani A., Thiagalingam S. (2025). Therapeutic Horizons: Gut Microbiome, Neuroinflammation, and Epigenetics in Neuropsychiatric Disorders. Cells.

[65] Gyarmati P., Song Y., Dotimas J., Yoshiba G., Christison A. (2021). Cross-sectional Comparisons of Gut Microbiome and Short-chain Fatty Acid Levels among Children with Varied Weight Classifications. Pediatr. Obes..

[66] Morgado M.C., Sousa M., Coelho A.B., Costa J.A., Seabra A. (2023). Exploring Gut Microbiota and the Influence of Physical Activity Interventions on Overweight and Obese Children and Adolescents: A Systematic Review. Healthcare.

[67] Musso G., Gambino R., Cassader M. (2010). Obesity, Diabetes, and Gut Microbiota. Diabetes Care.

[68] Akagbosu C.O., Nadler E.P., Levy S., Hourigan S.K. (2022). The Role of the Gut Microbiome in Pediatric Obesity and Bariatric Surgery. Int. J. Mol. Sci..

[69] Del Chierico F., Abbatini F., Russo A., Quagliariello A., Reddel S., Capoccia D., Caccamo R., Ginanni Corradini S., Nobili V., De Peppo F. (2018). Gut Microbiota Markers in Obese Adolescent and Adult Patients: Age-Dependent Differential Patterns. Front. Microbiol..

[70] Indiani C.M.D.S.P., Rizzardi K.F., Castelo P.M., Ferraz L.F.C., Darrieux M., Parisotto T.M. (2018). Childhood Obesity and Firmicutes/Bacteroidetes Ratio in the Gut Microbiota: A Systematic Review. Child. Obes..

[71] Wang L., Yi Q., Xu H., Liu H., Tan B., Deng H., Chen Y., Wang R., Tang F., Cheng X. (2024). Alterations in the Gut Microbiota Community Are Associated with Childhood Obesity and Precocious Puberty. BMC Microbiol..

[72] Turnbaugh P.J., Hamady M., Yatsunenko T., Cantarel B.L., Duncan A., Ley R.E., Sogin M.L., Jones W.J., Roe B.A., Affourtit J.P. (2009). A Core Gut Microbiome in Obese and Lean Twins. Nature.

[73] Bäckhed F., Ding H., Wang T., Hooper L.V., Koh G.Y., Nagy A., Semenkovich C.F., Gordon J.I. (2004). The Gut Microbiota as an Environmental Factor That Regulates Fat Storage. Proc. Natl. Acad. Sci. USA.

[74] Brestoff J.R., Artis D. (2015). Immune Regulation of Metabolic Homeostasis in Health and Disease. Cell.

[75] Tarantino G. (2014). Gut Microbiome, Obesity-Related Comorbidities, and Low-Grade Chronic Inflammation. J. Clin. Endocrinol. Metab..

[76] Da Silva T.F., Casarotti S.N., De Oliveira G.L.V., Penna A.L.B. (2021). The Impact of Probiotics, Prebiotics, and Synbiotics on the Biochemical, Clinical, and Immunological Markers, as Well as on the Gut Microbiota of Obese Hosts. Crit. Rev. Food Sci. Nutr..

[77] Granato A., Xie Q.Y., Wong A., Yau C., Noseworthy R., Chen T., Gianetto-Hill C., Allen-Vercoe E., Guidos C.J., Hamilton J.K. (2025). Metabolic Dysfunction Associated with Alterations in Gut Microbiota in Adolescents with Obesity. Diabetes.

[78] Cao B., Sun Y., Lam C., Chen Y., Dri C.E., McIntyre R.S. (2025). Interaction between Dietary Omega-3 Polyunsaturated Fatty Acids, Obesity and Gut Microbiota in Preclinical Models: A Systematic Review of Randomized Controlled Trials. Diabetes Obes. Metab..

[79] Xia Z., Li Y., Yin J., Gong Z., Sun J., Shen S., Yang Y., Liu T., Wang L., Huo J. (2025). Integrating Metabolomics and Gut Microbiota to Identify Key Biomarkers and Regulatory Pathways Underlying Metabolic Heterogeneity in Childhood Obesity. Nutrients.

[80] Chen Y., Tilves C., Bohn B., Doyon M., Bouchard L., Perron P., Guerin R., Masse E., Hivert M., Mueller N.T. (2025). Gut Microbiota and Microbial Metabolites Are Associated with Body Composition in 5-year-old Children: A Cross-sectional Study in the Gen3G Cohort. Pediatr. Obes..

[81] Chekima K., Yan S.W., Lee S.W.H., Wong T.Z., Noor M.I., Ooi Y.B., Metzendorf M.-I., Lai N.M. (2023). Low Glycaemic Index or Low Glycaemic Load Diets for People with Overweight or Obesity. Cochrane Database Syst. Rev..

[82] Martínez-González M.A., Gea A., Ruiz-Canela M. (2019). The Mediterranean Diet and Cardiovascular Health: A Critical Review. Circ. Res..

[83] De Filippis F., Pellegrini N., Vannini L., Jeffery I.B., La Storia A., Laghi L., Serrazanetti D.I., Di Cagno R., Ferrocino I., Lazzi C. (2016). High-Level Adherence to a Mediterranean Diet Beneficially Impacts the Gut Microbiota and Associated Metabolome. Gut.

[84] Myhrstad M.C.W., Tunsjø H., Charnock C., Telle-Hansen V.H. (2020). Dietary Fiber, Gut Microbiota, and Metabolic Regulation—Current Status in Human Randomized Trials. Nutrients.

[85] Makki K., Deehan E.C., Walter J., Bäckhed F. (2018). The Impact of Dietary Fiber on Gut Microbiota in Host Health and Disease. Cell Host Microbe.

[86] Perrone P., D’Angelo S. (2025). Gut Microbiota Modulation Through Mediterranean Diet Foods: Implications for Human Health. Nutrients.

[87] Khavandegar A., Heidarzadeh A., Angoorani P., Hasani-Ranjbar S., Ejtahed H.-S., Larijani B., Qorbani M. (2024). Adherence to the Mediterranean Diet Can Beneficially Affect the Gut Microbiota Composition: A Systematic Review. BMC Med. Genom..

[88] Ríos-Covián D., Ruas-Madiedo P., Margolles A., Gueimonde M., De Los Reyes-Gavilán C.G., Salazar N. (2016). Intestinal Short Chain Fatty Acids and Their Link with Diet and Human Health. Front. Microbiol..

[89] Koh A., De Vadder F., Kovatcheva-Datchary P., Bäckhed F. (2016). From Dietary Fiber to Host Physiology: Short-Chain Fatty Acids as Key Bacterial Metabolites. Cell.

[90] Chambers E.S., Byrne C.S., Rugyendo A., Morrison D.J., Preston T., Tedford C., Bell J.D., Thomas L., Akbar A.N., Riddell N.E. (2019). The Effects of Dietary Supplementation with Inulin and Inulin-propionate Ester on Hepatic Steatosis in Adults with Non-alcoholic Fatty Liver Disease. Diabetes Obes. Metab..

[91] Serrano-Gómez G., Yañez F., Soler Z., Pons-Tarin M., Mayorga L., Herrera-deGuise C., Borruel N., Rodriguez-Sinovas A., Consegal M., Manjón I. (2025). Microbiome Multi-Omics Analysis Reveals Novel Biomarkers and Mechanisms Linked with CD Etiopathology. Biomark. Res..

[92] Serra-Majem L., Román-Viñas B., Sanchez-Villegas A., Guasch-Ferré M., Corella D., La Vecchia C. (2019). Benefits of the Mediterranean Diet: Epidemiological and Molecular Aspects. Mol. Asp. Med..

[93] Zambrano A.K., Cadena-Ullauri S., Ruiz-Pozo V.A., Tamayo-Trujillo R., Paz-Cruz E., Guevara-Ramírez P., Frias-Toral E., Simancas-Racines D. (2024). Impact of Fundamental Components of the Mediterranean Diet on the Microbiota Composition in Blood Pressure Regulation. J. Transl. Med..

[94] Garicano Vilar E., López Oliva S., Penadés B.F., Sánchez Niño G.M., Terrén Lora A., Sanz Rojo S., Mauro Martín I.S. (2024). Mediterranean Diet Effect on the Intestinal Microbiota, Symptoms, and Markers in Patients with Functional Gastrointestinal Disorders. Microorganisms.

[95] Zheng X., Zhang W., Wan X., Lv X., Lin P., Si S., Xue F., Wang A., Cao Y. (2024). The Effects of Mediterranean Diet on Cardiovascular Risk Factors, Glycemic Control and Weight Loss in Patients with Type 2 Diabetes: A Meta-Analysis. BMC Nutr..

[96] Qu C., Zhao J., Lai J., Wu X., Huang P., Zhu T., Li Y., Liu T., Yuan J., Wang N. (2024). Adherence to a Mediterranean Diet Is Associated with a Lower Risk of Diabetic Kidney Disease among Individuals with Hyperglycemia: A Prospective Cohort Study. BMC Med..

[97] Sebastian S.A., Padda I., Johal G. (2024). Long-Term Impact of Mediterranean Diet on Cardiovascular Disease Prevention: A Systematic Review and Meta-Analysis of Randomized Controlled Trials. Curr. Probl. Cardiol..

[98] López-Gil J.F., García-Hermoso A., Martínez-González M.Á., Rodríguez-Artalejo F. (2024). Mediterranean Diet and Cardiometabolic Biomarkers in Children and Adolescents: A Systematic Review and Meta-Analysis. JAMA Netw. Open.

[99] Yurtdaş G., Akbulut G., Baran M., Yılmaz C. (2022). The Effects of Mediterranean Diet on Hepatic Steatosis, Oxidative Stress, and Inflammation in Adolescents with non-alcoholic Fatty Liver Disease: A Randomized Controlled Trial. Pediatr. Obes..

[100] Blancas-Sánchez I.M., Del Rosal Jurado M., Aparicio-Martínez P., Quintana Navarro G., Vaquero-Abellan M., Castro Jiménez R.A., Fonseca Pozo F.J. (2022). A Mediterranean-Diet-Based Nutritional Intervention for Children with Prediabetes in a Rural Town: A Pilot Randomized Controlled Trial. Nutrients.

[101] Hill C., Guarner F., Reid G., Gibson G.R., Merenstein D.J., Pot B., Morelli L., Canani R.B., Flint H.J., Salminen S. (2014). The International Scientific Association for Probiotics and Prebiotics Consensus Statement on the Scope and Appropriate Use of the Term Probiotic. Nat. Rev. Gastroenterol. Hepatol..

[102] Zhang L., Wang F., Wang R., Sun B., Liu P.J. (2024). Effects of Probiotics, Prebiotics, and Synbiotics on Cardiometabolic Risk Factors in Children and Adolescents with Overweight or Obesity: A Systematic Review and Bayesian Network Meta-Analysis. Crit. Rev. Food Sci. Nutr..

[103] Cox A.J., West N.P., Cripps A.W. (2015). Obesity, Inflammation, and the Gut Microbiota. Lancet Diabetes Endocrinol..

[104] Amat-Bou M., Garcia-Ribera S., Climent E., Piquer-Garcia I., Corripio R., Sanchez-Infantes D., Villalta L., Elias M., Jiménez-Chillarón J.C., Chenoll E. (2020). Effects of Bifidobacterium Animalis Subsp. Lactis (BPL1) Supplementation in Children and Adolescents with Prader-Willi Syndrome: A Randomized Crossover Trial. Nutrients.

[105] Loy M.H., Usseglio J., Lasalandra D., Gold M.A. (2023). Probiotic Use in Children and Adolescents with Overweight or Obesity: A Scoping Review. Child. Obes..

[106] Vajro P., Mandato C., Licenziati M.R., Franzese A., Vitale D.F., Lenta S., Caropreso M., Vallone G., Meli R. (2011). Effects of Lactobacillus Rhamnosus Strain GG in Pediatric Obesity-Related Liver Disease. J. Pediatr. Gastroenterol. Nutr..

[107] López-Moreno A., Suárez A., Avanzi C., Monteoliva-Sánchez M., Aguilera M. (2020). Probiotic Strains and Intervention Total Doses for Modulating Obesity-Related Microbiota Dysbiosis: A Systematic Review and Meta-Analysis. Nutrients.

[108] Jones R.B., Alderete T.L., Martin A.A., Geary B.A., Hwang D.H., Palmer S.L., Goran M.I. (2018). Probiotic Supplementation Increases Obesity with No Detectable Effects on Liver Fat or Gut Microbiota in Obese Hispanic Adolescents: A 16-Week, Randomized, Placebo-Controlled Trial. Pediatr. Obes..

[109] Luzzi A., Briata I.M., Di Napoli I., Giugliano S., Di Sabatino A., Rescigno M., Cena H. (2024). Prebiotics, Probiotics, Synbiotics and Postbiotics to Adolescents in Metabolic Syndrome. Clin. Nutr..

[110] Wieërs G., Belkhir L., Enaud R., Leclercq S., Philippart de Foy J.-M., Dequenne I., de Timary P., Cani P.D. (2019). How Probiotics Affect the Microbiota. Front. Cell. Infect. Microbiol..

[111] Ma T., Shen X., Shi X., Sakandar H.A., Quan K., Li Y., Jin H., Kwok L.-Y., Zhang H., Sun Z. (2023). Targeting Gut Microbiota and Metabolism as the Major Probiotic Mechanism-An Evidence-Based Review. Trends Food Sci. Technol..

[112] Crovesy L., Ostrowski M., Ferreira D.M.T.P., Rosado E.L., Soares-Mota M. (2017). Effect of Lactobacillus on Body Weight and Body Fat in Overweight Subjects: A Systematic Review of Randomized Controlled Clinical Trials. Int. J. Obes..

[113] Ejtahed H.-S., Angoorani P., Soroush A.-R., Atlasi R., Hasani-Ranjbar S., Mortazavian A.M., Larijani B. (2019). Probiotics Supplementation for the Obesity Management; A Systematic Review of Animal Studies and Clinical Trials. J. Funct. Foods.

[114] Choi W.J., Dong H.J., Jeong H.U., Jung H.H., Kim Y.-H., Kim T.H. (2019). Antiobesity Effects of Lactobacillus Plantarum LMT1-48 Accompanied by Inhibition of Enterobacter Cloacae in the Intestine of Diet-Induced Obese Mice. J. Med. Food.

[115] Choi W.J., Dong H.J., Jeong H.U., Ryu D.W., Song S.M., Kim Y.R., Jung H.H., Kim T.H., Kim Y.-H. (2020). Lactobacillus Plantarum LMT1-48 Exerts Anti-Obesity Effect in High-Fat Diet-Induced Obese Mice by Regulating Expression of Lipogenic Genes. Sci. Rep..

[116] Sohn M., Jung H., Lee W.S., Kim T.H., Lim S. (2023). Effect of Lactobacillus Plantarum LMT1-48 on Body Fat in Overweight Subjects: A Randomized, Double-Blind, Placebo-Controlled Trial. Diabetes Metab. J..

[117] Lee S.-B., Yoo B., Baeg C., Yun J., Ryu D., Kim G., Kim S., Shin H., Lee J.H. (2025). A 12-Week, Randomized, Double-Blind, Placebo-Controlled Study to Evaluate the Efficacy and Safety of Lactobacillus Plantarum LMT1-48 on Body Fat Loss. Nutrients.

[118] Solito A., Bozzi Cionci N., Calgaro M., Caputo M., Vannini L., Hasballa I., Archero F., Giglione E., Ricotti R., Walker G.E. (2021). Supplementation with Bifidobacterium Breve BR03 and B632 Strains Improved Insulin Sensitivity in Children and Adolescents with Obesity in a Cross-over, Randomized Double-Blind Placebo-Controlled Trial. Clin. Nutr..

[119] Sung H.K., Youn S.J., Choi Y., Eun S.W., Shin S.M. (2022). Body Fat Reduction Effect of Bifidobacterium Breve B-3: A Randomized, Double-Blind, Placebo Comparative Clinical Trial. Nutrients.

[120] Minami J., Iwabuchi N., Tanaka M., Yamauchi K., Xiao J., Abe F., Sakane N. (2018). Effects of *Bifidobacterium Breve* B-3 on Body Fat Reductions in Pre-Obese Adults: A Randomized, Double-Blind, Placebo-Controlled Trial. Biosci. Microbiota Food Health.

[121] Kondo S., Xiao J., Satoh T., Odamaki T., Takahashi S., Sugahara H., Yaeshima T., Iwatsuki K., Kamei A., Abe K. (2010). Antiobesity Effects of *Bifidobacterium Breve* Strain B-3 Supplementation in a Mouse Model with High-Fat Diet-Induced Obesity. Biosci. Biotechnol. Biochem..

[122] Kondo S., Kamei A., Xiao J.Z., Iwatsuki K., Abe K. (2013). Bifidobacterium Breve B-3 Exerts Metabolic Syndrome-Suppressing Effects in the Liver of Diet-Induced Obese Mice: A DNA Microarray Analysis. Benef. Microbes.

[123] Minami J., Kondo S., Yanagisawa N., Odamaki T., Xiao J., Abe F., Nakajima S., Hamamoto Y., Saitoh S., Shimoda T. (2015). Oral Administration of *Bifidobacterium Breve* B-3 Modifies Metabolic Functions in Adults with Obese Tendencies in a Randomised Controlled Trial. J. Nutr. Sci..

[124] Rodrigo T., Dulani S., Nimali Seneviratne S., De Silva A.P., Fernando J., De Silva H.J., Jayasekera, Wickramasinghe V.P. (2022). Effects of Probiotics Combined with Dietary and Lifestyle Modification on Clinical, Biochemical, and Radiological Parameters in Obese Children with Nonalcoholic Fatty Liver Disease/Nonalcoholic Steatohepatitis: A Randomized Clinical Trial. Clin. Exp. Pediatr..

[125] Chen A.-C., Fang T.-J., Ho H.-H., Chen J.-F., Kuo Y.-W., Huang Y.-Y., Tsai S.-Y., Wu S.-F., Lin H.-C., Yeh Y.-T. (2022). A Multi-Strain Probiotic Blend Reshaped Obesity-Related Gut Dysbiosis and Improved Lipid Metabolism in Obese Children. Front. Nutr..

[126] Li Y., Liu T., Qin L., Wu L. (2023). Effects of Probiotic Administration on Overweight or Obese Children: A Meta-Analysis and Systematic Review. J. Transl. Med..

[127] Gibson G.R., Hutkins R., Sanders M.E., Prescott S.L., Reimer R.A., Salminen S.J., Scott K., Stanton C., Swanson K.S., Cani P.D. (2017). Expert Consensus Document: The International Scientific Association for Probiotics and Prebiotics (ISAPP) Consensus Statement on the Definition and Scope of Prebiotics. Nat. Rev. Gastroenterol. Hepatol..

[128] Liber A., Szajewska H. (2014). Effect of Oligofructose Supplementation on Body Weight in Overweight and Obese Children: A Randomised, Double-Blind, Placebo-Controlled Trial. Br. J. Nutr..

[129] Shinozaki K., Okuda M., Sasaki S., Kunitsugu I., Shigeta M. (2015). Dietary Fiber Consumption Decreases the Risks of Overweight and Hypercholesterolemia in Japanese Children. Ann. Nutr. Metab..

[130] Yang Z., Yang M., Deehan E.C., Cai C., Madsen K.L., Wine E., Li G., Li J., Liu J., Zhang Z. (2024). Dietary Fiber for the Prevention of Childhood Obesity: A Focus on the Involvement of the Gut Microbiota. Gut Microbes.

[131] Fiore G., Magenes V.C., DI Profio E., Milanta C., Calcaterra V., Diamanti A., Campoy C., Zuccotti G., Verduci E. (2022). Gut Microbiota in Obesity and Related Comorbidities in Children and Adolescents: The Role of Biotics in Treatment. Minerva Pediatr..

[132] Antoniotti V., Partenope C., Solito A., Mancioppi V., Baima J., Medina F., Dimarakis S., Agostini A., Sista M.T., Monzani A. (2025). Efficacy of Myo-Inositol and Zinc on Insulin Resistance in a Paediatric Population with Obesity. Diabetes Obes. Metab..

[133] Wang Y., Salonen A., Jian C. (2023). Can Prebiotics Help Tackle the Childhood Obesity Epidemic?. Front. Endocrinol..

[134] Nicolucci A.C., Hume M.P., Martínez I., Mayengbam S., Walter J., Reimer R.A. (2017). Prebiotics Reduce Body Fat and Alter Intestinal Microbiota in Children Who Are Overweight or With Obesity. Gastroenterology.

[135] Hume M.P., Nicolucci A.C., Reimer R.A. (2017). Prebiotic Supplementation Improves Appetite Control in Children with Overweight and Obesity: A Randomized Controlled Trial1, 2, 3. Am. J. Clin. Nutr..

[136] Visuthranukul C., Kwanbunbumpen T., Chongpison Y., Chamni S., Panichsillaphakit E., Uaariyapanichkul J., Maholarnkij S., Chomtho S. (2022). The Impact of Dietary Fiber as a Prebiotic on Inflammation in Children with Obesity. Foods.

[137] Tayebi S.M., Poorhabibi H., Heidary D., Amini M.A., Sadeghi A. (2025). Impact of Aerobic Exercise on Chronic Inflammation in Older Adults: A Systematic Review and Meta-Analysis. BMC Sports Sci. Med. Rehabil..

[138] Hennigar S.R., McClung J.P., Pasiakos S.M. (2017). Nutritional Interventions and the IL-6 Response to Exercise. FASEB J..

[139] Ostrowski K., Schjerling P., Pedersen B.K. (2000). Physical Activity and Plasma Interleukin-6 in Humans—Effect of Intensity of Exercise. Eur. J. Appl. Physiol..

[140] Hojman P., Brolin C., Nørgaard-Christensen N., Dethlefsen C., Lauenborg B., Olsen C.K., Åbom M.M., Krag T., Gehl J., Pedersen B.K. (2019). IL-6 Release from Muscles during Exercise Is Stimulated by Lactate-Dependent Protease Activity. Am. J. Physiol.-Endocrinol. Metab..

[141] Visuthranukul C., Sriswasdi S., Tepaamorndech S., Chamni S., Leelahavanichkul A., Joyjinda Y., Aksornkitti V., Chomtho S. (2024). Enhancing Gut Microbiota and Microbial Function with Inulin Supplementation in Children with Obesity. Int. J. Obes..

[142] Holmes Z.C., Silverman J.D., Dressman H.K., Wei Z., Dallow E.P., Armstrong S.C., Seed P.C., Rawls J.F., David L.A. (2020). Short-Chain Fatty Acid Production by Gut Microbiota from Children with Obesity Differs According to Prebiotic Choice and Bacterial Community Composition. mBio.

[143] Swanson K.S., Gibson G.R., Hutkins R., Reimer R.A., Reid G., Verbeke K., Scott K.P., Holscher H.D., Azad M.B., Delzenne N.M. (2020). The International Scientific Association for Probiotics and Prebiotics (ISAPP) Consensus Statement on the Definition and Scope of Synbiotics. Nat. Rev. Gastroenterol. Hepatol..

[144] Yadav M., Sehrawat N., Sharma A.K., Kumar S., Singh R., Kumar A., Kumar A. (2024). Synbiotics as Potent Functional Food: Recent Updates on Therapeutic Potential and Mechanistic Insight. J. Food Sci. Technol..

[145] Barengolts E. (2016). Gut microbiota, prebiotics, probiotics, and synbiotics in management of obesity and prediabetes: Review of randomized controlled trials. Endocr. Pract..

[146] Markowiak P., Śliżewska K. (2017). Effects of Probiotics, Prebiotics, and Synbiotics on Human Health. Nutrients.

[147] Álvarez-Arraño V., Martín-Peláez S. (2021). Effects of Probiotics and Synbiotics on Weight Loss in Subjects with Overweight or Obesity: A Systematic Review. Nutrients.

[148] Kilic Yildirim G., Dinleyici M., Vandenplas Y., Dinleyici E.C. (2023). Effects of Synbiotic Supplementation on Intestinal Microbiota Composition in Children and Adolescents with Exogenous Obesity: (Probesity-2 Trial). Gut Pathog..

[149] Ipar N., Aydogdu S.D., Yildirim G.K., Inal M., Gies I., Vandenplas Y., Dinleyici E.C. (2015). Effects of Synbiotic on Anthropometry, Lipid Profile and Oxidative Stress in Obese Children. Benef. Microbes.

[150] Kianifar H.R., Ahanchian H., Safarian M., Javid A., Farsad-Naeimi A., Jafari S.A., kiani M.A., Dahri M. (2018). Effects of Synbiotics on Anthropometric Indices of Obesity in Children: A Randomized Double-Blind Placebo-Controlled Pilot Study. Top. Clin. Nutr..

[151] Mohammadi H., Ghavami A., Hadi A., Askari G., Symonds M., Miraghajani M. (2019). Effects of Pro-/Synbiotic Supplementation on Anthropometric and Metabolic Indices in Overweight or Obese Children and Adolescents: A Systematic Review and Meta-Analysis. Complement. Ther. Med..

[152] Atazadegan M.A., Heidari-Beni M., Entezari M.H., Sharifianjazi F., Kelishadi R. (2023). Effects of Synbiotic Supplementation on Anthropometric Indices and Body Composition in Overweight or Obese Children and Adolescents: A Randomized, Double-Blind, Placebo-Controlled Clinical Trial. World J. Pediatr..

[153] Vinderola G., Sanders M.E., Salminen S. (2022). The Concept of Postbiotics. Foods.

[154] Nazarinejad Z., Molani-Gol R., Roozbeh Nasiraie L., Ebrahimzadeh-Attari V. (2025). Postbiotics as a Novel Intervention for Obesity Management and Improving Metabolic Parameters: A Systematic Review and Meta-Analysis of Animal Studies. J. Transl. Med..

[155] Kovacs E., Szabo K., Varvara R.-A., Uifãlean A., Cozma A., Vulturar R., Sitar-Taut A.V., Gabbianelli R., Myhrstad M.C.W., Telle-Hansen V.H. (2025). Resistant Starch and Microbiota-Derived Secondary Metabolites: A Focus on Postbiotic Pathways in Gut Health and Irritable Bowel Syndrome. Int. J. Mol. Sci..

[156] Eslami M., Pakmehr A., Pourghazi F., Kami A., Ejtahed H.-S., Mohajeri-Tehrani M., Hasani-Ranjbar S., Larijani B. (2024). The Anti-Obesity Effects of Postbiotics: A Systematic Review of Pre-Clinical and Clinical Studies. Clin. Nutr. ESPEN.

[157] Han H., Xiong H., Liu Z., Liu X., Wang H., Kou J., Yi D., Shi Y., Wu H., Qiao J. (2025). Pasteurized Akkermansia Muciniphila Timepie001 Ameliorates DSS-Induced Ulcerative Colitis in Mice by Alleviating Intestinal Injury and Modulating Gut Microbiota. Front. Microbiol..

[158] Cavallari J.F., Fullerton M.D., Duggan B.M., Foley K.P., Denou E., Smith B.K., Desjardins E.M., Henriksbo B.D., Kim K.J., Tuinema B.R. (2017). Muramyl Dipeptide-Based Postbiotics Mitigate Obesity-Induced Insulin Resistance via IRF4. Cell Metab..

[159] Canfora E.E., Van Der Beek C.M., Jocken J.W.E., Goossens G.H., Holst J.J., Olde Damink S.W.M., Lenaerts K., Dejong C.H.C., Blaak E.E. (2017). Colonic Infusions of Short-Chain Fatty Acid Mixtures Promote Energy Metabolism in Overweight/Obese Men: A Randomized Crossover Trial. Sci. Rep..

[160] Leong K.S.W., O’Sullivan J.M., Derraik J.G.B., Cutfield W.S. (2020). Gut Microbiome Transfer—Finding the Perfect Fit. Clin. Endocrinol..

[161] Yadegar A., Bar-Yoseph H., Monaghan T.M., Pakpour S., Severino A., Kuijper E.J., Smits W.K., Terveer E.M., Neupane S., Nabavi-Rad A. (2024). Fecal Microbiota Transplantation: Current Challenges and Future Landscapes. Clin. Microbiol. Rev..

[162] Cymbal M., Chatterjee A., Baggott B. (2025). Fecal Microbiota Transplantation: Current Evidence and Future Directions. Clevel. Clin. J. Med..

[163] Del Chierico F., Manco M., Gardini S., Guarrasi V., Russo A., Bianchi M., Tortosa V., Quagliariello A., Shashaj B., Fintini D. (2021). Fecal Microbiota Signatures of Insulin Resistance, Inflammation, and Metabolic Syndrome in Youth with Obesity: A Pilot Study. Acta Diabetol..

[164] Nóbrega R., Costa C.F.F.A., Cerqueira Ó., Inês A., Carrola J.S., Gonçalves C. (2025). Association between Gut Microbiota and Pediatric Obesity: A Systematic Review. Nutrition.

[165] Zikou E., Koliaki C., Makrilakis K. (2024). The Role of Fecal Microbiota Transplantation (FMT) in the Management of Metabolic Diseases in Humans: A Narrative Review. Biomedicines.

[166] Yu E.W., Gao L., Stastka P., Cheney M.C., Mahabamunuge J., Torres Soto M., Ford C.B., Bryant J.A., Henn M.R., Hohmann E.L. (2020). Fecal Microbiota Transplantation for the Improvement of Metabolism in Obesity: The FMT-TRIM Double-Blind Placebo-Controlled Pilot Trial. PLoS Med..

[167] Kootte R.S., Levin E., Salojärvi J., Smits L.P., Hartstra A.V., Udayappan S.D., Hermes G., Bouter K.E., Koopen A.M., Holst J.J. (2017). Improvement of Insulin Sensitivity after Lean Donor Feces in Metabolic Syndrome Is Driven by Baseline Intestinal Microbiota Composition. Cell Metab..

[168] Leong K.S.W., Jayasinghe T.N., Wilson B.C., Derraik J.G.B., Albert B.B., Chiavaroli V., Svirskis D.M., Beck K.L., Conlon C.A., Jiang Y. (2020). Effects of Fecal Microbiome Transfer in Adolescents with Obesity: The Gut Bugs Randomized Controlled Trial. JAMA Netw. Open.

[169] Wang M., Zhang Z., Liu Y., Jian E., Ye P., Jiang H., Yu X., Cai P. (2024). Research Trends between Childhood Obesity and Gut Microbiota: A Bibliometric Analysis (2002–2023). Front. Microbiol..

[170] Fahim S.M., Huey S.L., Palma Molina X.E., Agarwal N., Ridwan P., Ji N., Kibbee M., Kuriyan R., Finkelstein J.L., Mehta S. (2025). Gut Microbiome-Based Interventions for the Management of Obesity in Children and Adolescents Aged up to 19 Years. Cochrane Database Syst. Rev..

[171] Dao M.C., Everard A., Aron-Wisnewsky J., Sokolovska N., Prifti E., Verger E.O., Kayser B.D., Levenez F., Chilloux J., Hoyles L. (2016). *Akkermansia Muciniphila* and Improved Metabolic Health during a Dietary Intervention in Obesity: Relationship with Gut Microbiome Richness and Ecology. Gut.

[172] Everard A., Belzer C., Geurts L., Ouwerkerk J.P., Druart C., Bindels L.B., Guiot Y., Derrien M., Muccioli G.G., Delzenne N.M. (2013). Cross-Talk between *Akkermansia Muciniphila* and Intestinal Epithelium Controls Diet-Induced Obesity. Proc. Natl. Acad. Sci. USA.

[173] Wilson B.C., Zuppi M., Derraik J.G.B., Albert B.B., Tweedie-Cullen R.Y., Leong K.S.W., Beck K.L., Vatanen T., O’Sullivan J.M., Cutfield W.S. (2025). Long-Term Health Outcomes in Adolescents with Obesity Treated with Faecal Microbiota Transplantation: 4-Year Follow-Up. Nat. Commun..

[174] Zou B., Liu S.-X., Li X.-S., He J.-Y., Dong C., Ruan M.-L., Xu L., Bai T., Huang Z.-H., Shu S.-N. (2022). Long-Term Safety and Efficacy of Fecal Microbiota Transplantation in 74 Children: A Single-Center Retrospective Study. Front. Pediatr..

[175] Conover K.R., Absah I., Ballal S., Brumbaugh D., Cho S., Cardenas M.C., Knackstedt E.D., Goyal A., Jensen M.K., Kaplan J.L. (2023). Fecal Microbiota Transplantation for *Clostridioides difficile* Infection in Immunocompromised Pediatric Patients. J. Pediatr. Gastroenterol. Nutr..

[176] Neyrinck A.M., Ahmed H., Leyrolle Q., Leclercq S., Amadieu C., Meuronen T., Layé S., Cani P.D., Kärkkäinen O., Bindels L.B. (2025). Fecal Transplantation from Humans with Obesity to Mice Drives a Selective Microbial Signature without Impacting Behavioral and Metabolic Health. Sci. Rep..

[177] Porcari S., Benech N., Valles-Colomer M., Segata N., Gasbarrini A., Cammarota G., Sokol H., Ianiro G. (2023). Key Determinants of Success in Fecal Microbiota Transplantation: From Microbiome to Clinic. Cell Host Microbe.

[178] Lai Z.-L., Tseng C.-H., Ho H.J., Cheung C.K.Y., Lin J.-Y., Chen Y.-J., Cheng F.-C., Hsu Y.-C., Lin J.-T., El-Omar E.M. (2018). Fecal Microbiota Transplantation Confers Beneficial Metabolic Effects of Diet and Exercise on Diet-Induced Obese Mice. Sci. Rep..

[179] Bhatia Z., Kumar S., Seshadri S. (2024). Fecal Microbiota Transplantation as a Potential Therapeutic Approach to Improve Impaired Glucose Tolerance via Gut Microbiota Modulation in Rat Model. J. Diabetes. Metab. Disord..

[180] Yang Y., Cui G., Qian J., Xu Y., Li B., Shi Y., Le G., Xie Y. (2024). Fecal Microbiota Transplantation from Methionine-Restricted Diet Mouse Donors Reduces Fat Deposition in Obese Mice by Remodeling the Gut Microbiota. Food Biosci..

[181] Mao X., Larsen S.B., Zachariassen L.S.F., Brunse A., Adamberg S., Mejia J.L.C., Larsen F., Adamberg K., Nielsen D.S., Hansen A.K. (2024). Transfer of Modified Gut Viromes Improves Symptoms Associated with Metabolic Syndrome in Obese Male Mice. Nat. Commun..

[182] Wenjiao D., Yurou W., Jiaqi X., Yan H., Hongfang J., Min C., Jianjin G. (2025). Animal Studies on the Modulation of Differential Efficacy of Polyethylene Glycol Loxenatide by Intestinal Flora. Front. Endocrinol..

[183] Chen Q., Wu C., Xu J., Ye C., Chen X., Tian H., Zong N., Zhang S., Li L., Gao Y. (2024). Donor-Recipient Intermicrobial Interactions Impact Transfer of Subspecies and Fecal Microbiota Transplantation Outcome. Cell Host Microbe.

[184] Keubler L.M., Talbot S.R., Bleich A., Boyle E.C. (2023). Systematic Review and Meta-Analysis of the Effect of Fecal Microbiota Transplantation on Behavior in Animals. Neurosci. Biobehav. Rev..

[185] Le Roy T., Debédat J., Marquet F., Da-Cunha C., Ichou F., Guerre-Millo M., Kapel N., Aron-Wisnewsky J., Clément K. (2019). Comparative Evaluation of Microbiota Engraftment Following Fecal Microbiota Transfer in Mice Models: Age, Kinetic and Microbial Status Matter. Front. Microbiol..

[186] Hou K., Wu Z.-X., Chen X.-Y., Wang J.-Q., Zhang D., Xiao C., Zhu D., Koya J.B., Wei L., Li J. (2022). Microbiota in Health and Diseases. Signal Transduct. Target. Ther..

[187] Liu Y., Hu Y., Ma B., Wang Z., Wei B. (2025). Gut Microbiota and Exercise: Probiotics to Modify the Composition and Roles of the Gut Microbiota in the Context of 3P Medicine. Microb. Ecol..

